# Towards a Quality Care Climate Perspective: A Systematic Review of Associations Among Patient Experience, Patient Outcomes, and Organisational Climate Factors in Hospitals

**DOI:** 10.3390/ijerph23020268

**Published:** 2026-02-20

**Authors:** Seth Ayisi Addo, Reidar Johan Mykletun, Espen Olsen

**Affiliations:** 1 Unit of Occupational Medicine, Institute of Environmental Medicine, Karolinska Institutet, Nobels Väg 13, Box 210, 171 77 Stockholm, Sweden; 2 Department of Innovation, Leadership, and Marketing, University of Stavanger Business School and Law, 4036 Stavanger, Norway; reidar.j.mykletun@uis.no (R.J.M.);

**Keywords:** patient experience, patient outcomes, organisational climate factors, systematic review, quality care, hospital

## Abstract

Objective (study question): The main purpose of this systematic review was to conduct a qualitative synthesis of quantitative studies among patient experience, patient outcomes, and organisational climate factors. The review sought to answer the following research questions: (i) What are the main directions, dominant methods, and theories on the associations among these concepts? (ii) What theoretical propositions can be made? Data sources/study setting (w/hospital/institution setting anonymised): The study involved a search for literature in PubMed, PsychINFO, Medline, CINAHL, Academic Search Premier, Scopus, and Web of Science between 2007 and 2022 with the guidance of a librarian. The search was limited to English language and to human adult inpatients. Study design: This study primarily employed a systematic review method, following the guidelines in the PRISMA statement. Data collection/extraction methods: Articles were screened and excluded first on title and abstract, and then on fulltexts. Quality assessments were done on remaining articles using the National Institutes of Health (NIH) quality assessment tool for observational, cohort and cross-sectional studies. Data was extracted from articles that met the inclusion criteria and passed the checks. Principal findings: The search identified 11,571 records that were checked for duplications. After removing duplicates and applying the eligibility criteria, a final list of 220 articles were included for the qualitative synthesis. Results showed that the relationships among the concepts were more conclusive and generally positive rather than negative, especially between patient experience and patient outcomes. The review, however, showed areas that required more attention such as an encompassing quality-oriented care climate theory, validation of patient-reported instruments, and longitudinal studies linking subjective patient outcomes to objective patient outcomes. Conclusions: The review shows that conclusions can be drawn on the relationships among the variables, indicating that favourable factors in the hospitals, as perceived by patients, have positive implications for patient experiences and their outcomes. Based on this, an argument for an encompassing framework on quality care climate from the patients’ perspectives was made to enhance understanding of these relationships. Limitations: Among others, this review is limited by the search restriction to quantitative studies or studies that employed mainly quantitative tools to assess associations or changes. Funding: This study received no external funding. Registration: PROSPERO ID- CRD42021291787.

## 1. Introduction and Rationale

Patient experience is defined as the individual and collective events and occurrences that are manifest in the caregiving process, seen to be influenced by expectations of patients, prior to receiving healthcare [1]. The concept is deemed as an essential and well-recognised indicator for assessing hospital performance [2], and it is considered by the World Health Organisation (WHO) as one of the determinants of quality healthcare [3]. The increasing attention on patient experience has also yielded research on its relationships with patient outcomes such as patient satisfaction [4,5,6,7]. Since the hospital is the framework within which healthcare is given to patients, the hospital environment (tangible and intangible) is likely to account for variations in experiences of patients as well as in patient outcomes. In order to enhance healthcare delivery, there is the need to account for the different but intersecting perspectives from patients and their providers [8]. It would therefore be disingenuous to assess patient experience and patient outcome relationships without considering the environment in which care is given, and how such factors may influence their outcomes.

Jones and Jenkins [9] maintained that the provision of health care services is the totality of interactions between healthcare professionals and patients occurring within an organisational and social context as well as an infrastructural system. This culminates into the organisational climate concept, defined as the measurable aspects of the work environment that are perceived and shared as the formal and informal practices, policies, and procedures by individuals (employees) in an organisation [10]. However, a systematic review conducted by MacDavitt, Chou, and Stone [11] on organisational climate and health care outcomes showed that associations between organisational climate factors and patient outcomes were inconsistent. This review was done using published articles between 1995 and 2007. Nonetheless, more recent research on organisational climate factors and patient variables such as outcomes and experiences have shown significant and positive associations [12,13,14,15]. MacDavitt et al. [11] concluded that evidence of associations between organisational climate and patient outcomes is not as robust as the one between organisational climate and nurse outcomes (e.g., job satisfaction and well-being), indicating a need for more research. Considering the years that have elapsed since the review by MacDavitt et al. [11], and the increasing attention on patient experience from researchers, there is the need for a systematic review to draw adequate conclusions on whether there have been some changes in the associations between organisational climate and patient-related variables, thus the rationale for the current study.

In a more recent, related review, Braithwaite, Herkes, Ludlow, Testa, and Lamprell [16] focused on associations between organisational culture and patient outcomes, encompassing the organisational climate concept within the broad concept of organisational culture. The current study, however, distinguishes between climate and culture, as done by MacDavitt et al. [11], and focuses on the former, and its associations with patient-reported variables. This study is a systematic review on the associations between patient experiences, patient outcomes such as patient safety and patient satisfaction as well as organisational climate factors such as hospital management/leadership, hospital systems and structure. The review focuses on both foundational climates, the broad workplace environment, and specific climates, such as safety climate and service climate [17]. The overarching goal is to make a descriptive synthesis of studies among the variables to draw informed conclusions on the general directions and theoretical underpinnings as well as propose a quality-oriented care climate framework. This review sought to answer the following questions:

1. What are the main directions, dominant methods, and theories on the associations among these concepts?
2. What relevant research and theoretical recommendations can be made?

### 1.1. Operational Definitions of Key Concepts and Search Framework

#### 1.1.1. Patient Experience

This review adopts the definition of patient experience by Wolf et al. [1] as individual and collective events and occurrences that are manifest in the caregiving process, seen to be influenced by expectations of patients, prior to receiving healthcare. Patient experience comprises different dimensions; notable amongst these are nurse services, doctor services, hospital standards, organisation, information, and communication, among others, seen in the plethora of patient-reported experience measures (PREMs) [18,19,20].

#### 1.1.2. Patient Outcomes

In this review, patient outcomes conceptually encompass all consequences and effects of the caregiving process on patients. These can either be objective (devoid of patients’ perceptions) such as mortality, injuries, adverse events, or subjective (based on patients’ perceptions) such as patient satisfaction, service quality, patient perceptions of safety, health benefits, etc. MacDavitt et al. [11] noted a lack of adequate studies examining objective patient outcomes attributable to lack of access to consistent data.

#### 1.1.3. Organisational Climate Factors

Although defined as perceptions of the formal and informal practices, policies, and procedures from employees [10], this review extends and operationalises this concept to encompass perceptions and experiences of both employees and patients in hospitals of the measurable aspects of the work environment. This review focuses on both foundational climate factors such as the hospital structure, leadership, and management as well as specific climate factors such as safety climate, service climate, etc.

**Figure 1:**
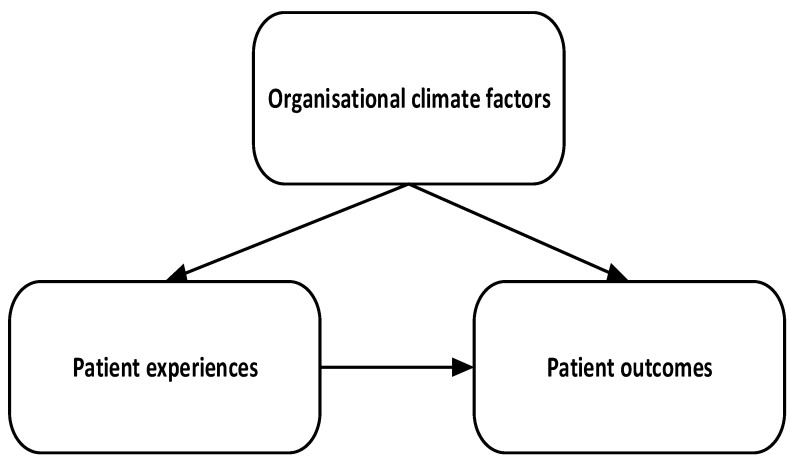
Search framework.

The review is guided by the framework in Figure 1, where organisational climate factors are seen to relate to both patient experiences/perceptions, and to patient outcomes. Patient experiences also relate to patient outcomes. These associations guided the general search for literature and inclusion. The framework also shows that overlaps among the concepts and their synonyms were captured in the search, which is quite common in the literature. For instance, it is common, although not correct, that some studies would intercha-nge patient experience and patient satisfaction.

### 1.2. The ‘Quality Care Climate’

Although studies abound on organisational climate, it remains a heavily contested concept. This contention has been based on its definitions. While some scholars define it as the interaction between an individual and the environment, others see it as a consequence of the ongoing behavioural practices in an organisation [21]. Another contention has also been about whether the concept should be unidimensional or multidimensional. Schneider [22] noted that due to the broad nature of the concept, the different units of analysis in organisations (e.g., individuals and teams) as well as the purpose of each inquiry, scholars have studied specific dimensions under climate such as safety climate and service climate [23,24]. Indeed, MacDavitt et al. [11] indicated that organisational climate had been measured in various ways, and perceptions of employees may not capture all aspects of the work environment adequately. Patients provide a complementary perspective to which organisational climate may be assessed since they experience aspects of the hospital environment. According to Nembhard, Northrup, Shaller, and Cleary [25], the absence of quality-oriented organisational climates have, in part, resulted in inadequate patient-centred care and quality in general. This begs the question: what should a quality-oriented climate be? The current study argues that a good conceptualisation and measurement of such a climate should mainly be from the patients’ perspective, since they are the centre of the caregiving process. This study uses the results of this review to propose the ‘quality care climate’ as a specific climate measured by patients’ assessments of hospital factors that underpin the organisational climate, their reported experiences of the caregiving process, as well as their objective and subjective outcomes.

## 2. Method

This review was conducted based on the guidelines in the Preferred Reporting Items for Systematic Reviews and Meta-Analyses (PRISMA) statement and checklists [26]. The checklists are presented as Supplementary Files in Supplementary A and Supplementary B. Prior to this, a protocol for this review was developed and registered in PROSPERO on 17 December 2021, with ID: CRD42021291787 [27] based on the Preferred Reporting Items for Systematic Review and Meta-Analysis Protocols (PRISMA-P) [28]. Apart from a change of risk-of-bias tool, no other major deviations from this study were recorded. The following specific steps were taken in completing this review: specifying eligibility criteria; search strategy and information sources; data management and screening; risk of bias and quality assessments; data extraction and qualitative/configurative synthesis.

### 2.1. Eligibility Criteria

This review included studies that focus on adult inpatients in general healthcare published in peer-reviewed journals from 2007 forward, due to the last review by MacDavitt et al. [11]. Studies were not restricted to any geographical location but only studies published in English were included. Studies employing quantitative methods or mixed methods to assess statistical associations (for instance in cross-sectional and longitudinal studies) or significant changes (for instance in studies using longitudinal, intervention, and RCT designs) among the variables were included. Lastly, studies that used primary or secondary responses from patients were included.

Studies that focused on children and childcare or on patients with one specific illness (e.g., stroke patients) were excluded. This was done to get a more nuanced and varied pool of studies for the review. The review also excluded studies focusing solely on patients in other departments in the hospital aside inpatient department (e.g., outpatient department, emergency department, etc.). It was believed that patients who are admitted to the hospital for days get ample time to assess their experiences of the hospital environment better. Lastly, studies that employ solely qualitative designs and methods were excluded as the review was interested in the general direction of associations among the variables. The authors decided that a review including qualitative designs and methods is better suited as a separate study to allow more room for discussion and nuance.

### 2.2. Search Strategy and Information Sources

A search string and strategy was developed by the researchers under the guidance of a librarian, taking the eligibility criteria into consideration. Synonyms for variables and search limits were developed based on the librarian’s advice. This search for literature was then carried out in APA PsycNET, PubMed, PsychINFO, Medline, CINAHL, Academic Search Premier, Web of Science, and Scopus. The search string was applied to and modified according to each database. The complete search string is provided in Supplementary C. The last date of search was 20 August 2022.

### 2.3. Predicting Variables and Outcome Variables

Patient experience and organisational climate factors were sought as predictors in studies. The main outcomes that were sought from studies were patient satisfaction, patient safety, service quality, and health benefits. These outcomes were prioritised because several studies have assessed their relationships with patient experiences. However, other studies that focus on relationships between patient experiences and other patient-related variables such as patient expectations were also included. Secondary outcomes also included patient adverse effects, harm, accidents recorded in hospitals, among others. Studies that presented patient experiences as outcomes of organisational climate factors were also included.

### 2.4. Data Management and Screening

**Figure 2:**
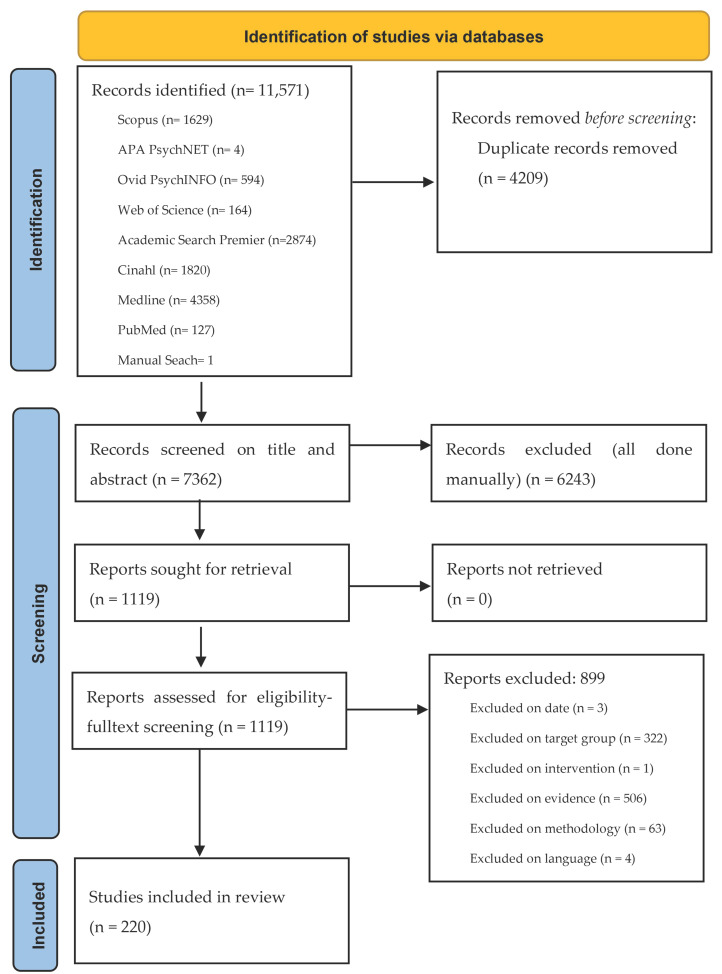
Flow diagram from literature identification to final inclusion.

The review team was made up of three researchers and two assistants. All team members had access to a shared account in the EPPI-Reviewer software, version 6 (United Kingdom) [29]. This software was used to screen articles after the search for literature, conduct risk of bias and quality assessments as well as data extraction. There were two screening phases: abstract and title screening, and fulltexts screening (see Figure 2 for details). The first phase was conducted by two researchers on each article, applying the inclusion criteria. The included articles after this phase were shared evenly among team members for the second screening phase. The research assistants were adequately trained by informing them of the purpose and eligibility criteria as well as in using EPPI. Any discrepancy on inclusion of an article was resolved through discussions among team members.

### 2.5. Risk of Bias and Overall Quality Assessments

After the second screening, a risk-of-bias assessment was conducted for each article included. Risk of bias was assessed by adapting questions from the National Institutes of Health (NIH) quality assessment tool for observational, cohort and cross-sectional studies [30]. The components of this tool include whether there were clearly stated objectives/questions, clearly specified population and justified sample, variations in levels of predictor variables, among others. Each question had three assessment options: yes, no, or cannot determine/not applicable/not reported. The tool also included an overall rating of the article as good, fair, or poor.

### 2.6. Data Extraction and Synthesis

Information from each article included in the final list was extracted and synthesised qualitatively. This approach was taken, instead of a meta-analysis, because the aim was to theoretically describe the overview of the relationships as a basis for a theoretical proposition. The extracted information comprised main purpose and variables, context, designs and sampling, main outcomes, etc. The review synthesis takes a configurative approach to describe the broad picture of the associations among the variables (similarities and differences), both theoretically and statistically. An association was determined positive, negative, or not significant based on the inferential statistics and results in a paper. To ensure robustness of synthesis, discussions on the themes were done among the researchers. This helped to boost the confidence and certainty in the synthesised information. Also, sensitivity was assessed by comparing overview of associations among the variables between studies conducted in the USA and those other than the USA.

## 3. Results

### 3.1. Study Identification

The search for literature identified a total of 11,571 records: APA PsycNET—4, PubMed—127, PsychINFO—594, Medline—4358, CINAHL—1820, Academic Search Premier—2874, Web of Science—164, Scopus—1629 and a manual search for 1 record. The titles and abstracts of these records were downloaded and imported into EPPI, where 4209 duplicates were found and excluded. Thus, 7362 records were eligible for screening. After title and abstract screening, 6243 records were excluded based on the eligibility criteria. For instance, if the article did not focus on adult inpatients, it was excluded based on target group, or if it employed qualitative methods for analysis, it was excluded based on methodology. The fulltexts of the remaining 1119 records were then downloaded, imported into EPPI, and screened. At this stage, 899 records were excluded, using the same eligibility criteria for the first screening, leaving a total of 220 for quality assessments and final inclusion. Figure 2 presents a workflow of the literature search and identification, screening, and final inclusion.

### 3.2. Study Characteristics

**Figure 3:**
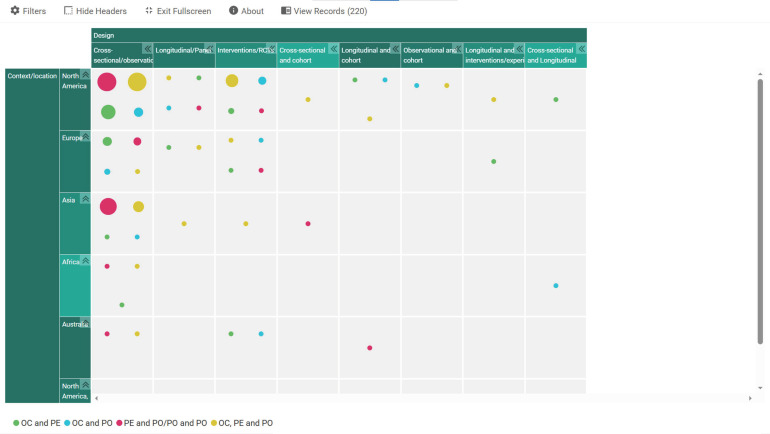
Snapshot of interactive mapping of study contexts, designs, and variables. OC: organisational climate; PE: patient experience; PO: patient outcomes.

Out of the 220 studies that were finally included, 118 of them were conducted primarily in the USA; 13 in China; 6 each in Canada, Norway, South Korea, Switzerland, and United Kingdom; 5 each in Australia and Germany; 4 each in Iran and Netherlands; 3 each in Indonesia, India, and Portugal; 2 each in Bangladesh, Ethiopia, Italy, Jordan, Pakistan, and Spain; and 1 each in Benin, Cyprus, Czech Republic, Faroe Islands, Ghana, Iraq, Ireland, Israel, Japan, Malaysia, Mexico, Nigeria, Poland, Saudi Arabia, Slovenia, Taiwan, Tunisia, and Turkey. There were two more in multiple countries, one conducted in Australia, Canada, New Zealand, United Kingdom, USA, Germany, and Netherlands, and the other conducted in 21 European countries. The studies were also characterised by the use of various designs: cross-sectional, longitudinal/repeated cross-sectional, cohort, observational, interventions, randomised control trials, as well as combinations of these designs. However, the majority of the studies (144) were cross-sectional studies. Data sources used were both primary and secondary comprising patient surveys, employee surveys, hospital data and records, etc. Notably, most of the studies in the USA employed secondary sources on patient experiences and satisfaction, staff perceptions as well as hospital records from annually gathered data that were publicly stored. Analytical approaches also ranged from various parametric to non-parametric tests. The studies also employed samples at individual and/or hospital, team, unit levels ranging from below 100 participants to thousands of participants. To give a detailed overview of the studies included, an interactive mapping of study contexts/locations against designs employed and segmented according to the variables under study was generated using EPPI. A snapshot of this is presented in Figure 3 while the full interactive version is presented as a link in a Supplementary File (Supplementary D).

### 3.3. Study Quality and Sensitivity Analysis

Each included article was assessed for quality using the risk-of bias-tools by two researchers independently. Based on the items, overall ratings were given for each article. There was about 90 percent agreement between the researchers after comparison. Discrepancies were then resolved together with the third researcher. Overall, 190 articles were rated as ‘Good’; 26 were rated as ‘Fair’; and 4 were rated as ‘Poor’. A sensitivity analysis was thus conducted to determine whether to include the articles rated poor. Based on this, the researchers agreed that they represented a small percentage of the entire number of included articles. More importantly, the analysis showed that these articles did not paint an entirely different picture from the better rated articles. It was concluded that these articles would not significantly change the direction or outcome of the review and therefore were still considered in the synthesis of results. The full table for the results of the risk analysis is presented as a Supplementary File (Supplementary E).

### 3.4. Synthesis of Studies

Similarities and differences were drawn from the variables and findings in the included studies and are presented under five broad themes: variables and factors, overview of associations, group comparisons, forms of interventions, and theoretical overview. A truncated version of the entire table containing the details of the extracted data for each study included is presented in Table 1. The full version of this table is presented as a Supplementary File (Supplementary F).

#### 3.4.1. Variables and Factors

First, organisational climate factors ranged from foundational issues such as care teams, nurse staffing, employee skills, hospital governance/ownership, and hospital size [31,32,33,34] to specific factors such as safety climate, civility climate, learning climate, among others [13,15,35,36,37]. These factors also ranged from tangible ones such as capacity/number of beds, hospital infrastructure, access to tablets, massage and music therapy, language assistance services, among others [38,39,40], to intangible ones such as teamwork, employee satisfaction, burnout, accreditation status, teaching status of hospitals, social environment quality, mergers, and acquisitions [41,42,43,44].

Secondly, experiences of patients were reported and measured on issues such as doctor and nurse communication, information sharing and education, discharge assistance services, perception of organisation and coordination among healthcare providers and teams, hospital structures and standards, and quality improvement programmes [4,45,46,47]. Regarding patient outcomes, studies ranged from subjective outcomes such as patient satisfaction, patient safety perception, patient ratings of service quality, patient loyalty, and patient trust [48,49,50] to objective outcomes such as patient falls, hospital mortality rate, mortality 30 days after discharge, patient harm and incidents, length of stay, hospital-acquired conditions (HACs), and other adverse events [51,52,53,54].

Notably, studies in countries that had comprehensive, annual, and publicly accessible data on nurse experiences, hospital factors, patient experiences and outcomes mainly employed these sources. For instance, most of the studies conducted in the USA used annual patient survey data of the Hospital Consumer Assessment of Health Providers and Systems (HCAHPS), developed by the Centers for Medicare & Medicaid Services (CMS), that gathers information from patients on their experiences, overall rating of hospital, willingness to return and willingness to recommend hospital [55,56]. This was also evident across studies in other countries such as Norway and China where annual data on patient variables were gathered [45,57,58].

In addition, some concepts were peculiar to some contexts. First, the concepts of magnet and non-magnet hospitals have been studied mainly in the USA [59,60]. Magnet hospitals are “those that have been designated as such by the American Nurses Credentialing Center (ANCC) because they have met specific criteria indicating they provide an excellent nursing work environment and the best care for patients” ([59], p. 22). This is an indication of quality in nursing. Second, the concepts of safety-net and non-safety-net hospitals have also been studied primarily in the USA [61]. Safety-net hospitals are defined as hospitals that have a large proportion of their patients being poor and vulnerable, and relying on state-sponsored healthcare to cover costs [61]. Lastly, the concepts of hospitalists and non-hospitalists were studied mainly in the USA [55,62]. The study of hospitalists and non-hospitalists was also studied in South Korea by Chae, Kim, Park, and Jang [63]. Hospitalists are generally understood as physicians (other than a patients’ primary care doctor) who are responsible for the care of hospitalised patients for the duration of the patient’s hospital stay [55,63]. What is notable with these concepts in the USA is that they are tied to hospital funding through the hospital value-based purchasing (VBP) using experience and satisfaction ratings of patients based on the HCAHPS by the CMS, a center focused on affordable healthcare for low-income patients.

#### 3.4.2. Overview of Associations

Generally, the relationships between organisational climate factors and patient-related factors are seemingly more positive than negative. Out of the 47 studies on organisational climate factors and patient experience, 32 of them found positive relationships or influences between favourable organisational climate factors and favourable patient experiences [64,65,66], 2 studies [61,67] found negative relationships, 3 studies [40,68,69] found no significant relationships/changes, while the remainder only assessed variances explained or comparisons [33].

Regarding organisational climate factors and patient outcomes, out of the 37 studies, 30 found positive relationships or influences between favourable organisational climate factors and favourable patient outcomes [13,41,42,51] but none recorded negative relationships, while 5 studies merely assessed explained variances and comparisons [70,71]. A study [72] found no significant effect of the implementation of an electronic dashboard on overall rapid response activations, deaths, unexpected intensive care unit (ICU) cases and cardiopulmonary arrests. Interestingly, a study by Tevis and Kennedy [73] found some paradoxical results. In their study, hospital structure in terms of high surgical volume related positively with more ICU cases, longer length of stay (LOS), more complications and higher readmission rates (objective outcomes) than lower surgical volume hospitals, but in contrast, higher surgical volume hospitals were likely to obtain higher patient satisfaction scores (subjective outcomes), regardless of the objective outcomes.

Notably, all 61 studies on patient experiences and patient outcomes mainly found positive relationships [31,45,46,74], with some partially reporting no significant relationships [75,76]. In addition, two studies [77,78] also linked subjective patient outcomes such as satisfaction to objective outcomes such as mortality and complications where results indicated positive relationships between subjective outcomes and favourable objective outcomes. For instance, Sacks et al. [78] found that hospitals rated higher on patient satisfaction recorded lower chances of 30-day mortality, failure to rescue and minor complications.

Furthermore, regarding studies that focused on all three concepts, out of the 73 studies, 52 found positive relationships or influences among favourable organisational climate factors, positive patient experiences and favourable patient outcomes [37,79,80,81], while none of them found negative relationships. Ten studies found partial non-significance [38,82,83]. For instance, Beauvais et al. [38] found significant relationships of age of plant with clinical care and patient experience but found no significant relationship of age of plant with patient safety and efficiency. The remaining 11 studies were mainly on comparisons between groups [53,55,84].

Lastly, inconsistencies were found particularly in studies on hospital/healthcare costs and patient experiences and outcomes. For instance, while some studies found that affordable hospital costs related negatively to patient experiences [61,67] or that higher hospital costs related positively with favourable patient outcomes and experiences [85], another study by Beauvais et al. [65] found that, on the contrary, higher hospitals’ prices and costs do not relate positively with patient experience and perceptions of quality. To elaborate, Liu et al. [67] assessed the relationship between Medicaid coverage (a USA state-sponsored affordable healthcare programme for citizens with very low incomes) and patient experiences. They found that the higher the proportion of Medicaid patients in a hospital, the lower the hospital’s rating on patient experience measures. This is in contrast to Beauvais et al.’s [65] assertion that higher pricing in comparison to costs might even relate with lower quality and patient experience in hospitals.

Considering that the majority of the studies were in the USA, the study authors decided to assess associations between the concepts in countries other than the USA. The overview showed a similar pattern with the majority of the studies reporting positive associations rather than negative, confirmed in the interactive mapping in Figure 3 (indicated earlier).

#### 3.4.3. Group Comparisons

Some studies also made comparisons between groups on levels of patient-reported variables. For instance, comparisons were made among public, private, and not-for-profit hospitals on patient outcomes, with private hospitals scoring better than public ones in more studies [32,86,87,88], between accredited and non-accredited hospitals or before and after accreditation, where accredited hospitals performed better [43,89].

Some studies also assessed differences in patient-reported variables based on hospital/unit sizes, with contradicting findings [58,90,91]. For instance, while Sjetne et al. [91] found that smaller hospitals (measured by number of beds) were rated higher on patient experiences than medium and larger hospitals, Hu et al. [58] found that larger hospitals (measured by number of beds and surgeries) scored better on patient experience and satisfaction than smaller hospitals.

Other studies also compared patient outcomes based on nursing excellence, that is, magnet versus non-magnet hospitals, with evidence to show that hospitals with higher nursing excellence (magnet) scored higher on patient outcomes [59,60,92]. Furthermore, Abor [32] made comparisons between different aspects of health governance and reported better quality of healthcare in hospitals with smaller board size as opposed to bigger board size, separate roles of CEO and board chair as opposed to a unified role, boards with more female representation as opposed to those with less, as well as boards that hold frequent meetings as opposed to boards that do not.

Comparisons were also made to show that hospitals in mergers and acquisitions scored lower on patient outcomes than their counterparts [53,84]; hospitalists performed better than non-hospitalists [55,62,63]; registered nurses scored better on patient satisfaction than nurse assistants but the latter have a comparative advantage in patient support [56]; and Catholic hospitals performed slightly better than non-Catholic hospitals [93].

Hospitals with stronger community orientation scored better on patient outcomes than those with weak orientations [94], whereas hospitals in rural locations scored lower on room cleanliness than those in urban locations [95]. Contradicting findings were seen between university or teaching hospitals versus non-university or non-teaching hospitals [91,96,97]. While Wray et al. [97] found that non-teaching hospitals performed better than general medicine teaching hospitals on experience and overall satisfaction, Nemati et al. [96] found that university hospitals scored better than non-university hospitals on patients’ perceptions of hospital service quality. Lastly, DiLeo, Borkowski, O’Connor, Datti, and Weech-Maldonado [98] compared Lesbian Gay Bisexual and Transgender (LGBT)-affiliated hospitals with non-affiliated hospitals and found that LGBT-affiliated hospitals performed better on patient experiences and outcomes than non-affiliated hospitals.

#### 3.4.4. Forms of Interventions

The studies using interventions and trials were geared towards improving experiences of patients with healthcare as well as patient and employee outcomes. The interventions took the forms of overlaps between tangible and intangible ones. The interventions included system, logistics and structure redesigns or upgrade with positive effects after implementation [99,100,101]. For instance, Kline et al. [100] studied the effects of a ward design and concluded that, after the move from a traditional medical ward to a newly developed ward, patient satisfaction and overall quality increased significantly.

There were interventions also on care coordination, collaboration, and communication [25,36,102,103,104]. Opper et al. [104] studied the effects of a health communication redesign. The intervention included daily interprofessional team rounding, in-room communication as well as nurse bedside shift report. The study found that after implementation, readmissions within 30 days and emergency department visits decreased significantly. Some interventions also focused on pre- and post-discharge care transitions with positive effects of the interventions [105,106,107], whereas Chan et al. [68] found no significant effects of a care transition intervention on patient experience measures.

Furthermore, the effects of bedside rounds interventions proved inconsistent [108,109,110]. For instance, while O’Leary et al. [110] found no significant differences between intervention group and control group on patient perceptions of shared decision making and satisfaction, Monash et al. [109] found that standardised attending rounds improved patient experience and satisfaction. Dunn et al. [108], however, found partial non-significance, where effects of interdisciplinary bedside rounds’ effects on length of stay were not significant but found a significant increase in the patient safety climate from the employees’ perspective after intervention.

Other interventions included electronic and IT such as electronic dashboard, electronic health records, and in-room webcams with favourable effects [72,111,112], safety intervention programmes [37,75,113], noise reduction [114], music and massage therapy with inconsistent findings [40,115], language assistance services [82,116], facial recognition [49], and patient relatives’ collaborations [74,117].

In most of these studies, the interventions achieved their desired effects on outcomes such as mortality, hospitalisation, patient experiences, patient satisfaction, 30-day readmission, hospital-acquired conditions, medication errors, and staff satisfaction. Out of 48 intervention studies, 38 of them found positive or favourable effects on outcomes [36,37,49] while 6 of them found no significant effects [40,52,68,110,118,119]. Three of the studies [25,108,120] found partial non-significant effects on outcomes. For instance, Hanskamp-Sebregts et al. [120] found that a patient safety auditing and feedback intervention did not have any significant effect on adverse events but showed a significant improvement in patient experiences and medication safety. The last study by Timmermans et al. [121] sought to assess the impact of physician assistants working together with doctors in comparison with a group of only doctors, finding that the doctor–physician mix scored higher on patient experience but found no significant difference in length of stay, quality and safety care. Although a few of these interventions had no significant effect on outcomes, none of the interventions achieved the reverse of the desired effects.

#### 3.4.5. Theoretical Overview

Most of the studies did not employ any underpinning theory. However, the majority of the studies that employed a theory used the Donabedian framework [122] for assessing healthcare quality [4,38,46,93]. The Donabedian framework makes inferences of quality under three categories: structure, process, and outcome. The structure deals with the setting in which care is given; the process deals with what is done in giving and receiving care; and the outcome is the effects of care on health and well-being [122,123]. A few studies also used the SERVQUAL model and expectancy-disconfirmation theory for assessing service quality gaps [32,124,125,126].

Other studies employed theories from organisation and management disciplines such as contingency theory [127], structural contingency theory [128], attribution theory [50], upper echelons theory of top managers [129], service fairness and equity theory [130], organisational theory [66], Meleis’ transitions theory [104], Swanson’s middle range theory of caring [131], resource dependency theory (RDT) and resource-based view (RBV) [98], voice of the customer [132], and nursing environment theory and theory of supportive design [133]. Self-developed conceptual frameworks were also used by some studies to illustrate the hypothesised relationships among variables or explain an adapted framework.

## 4. Discussion

This review sought to outline the main directions, dominant methods, and theories on the associations among the concepts under study. Resounding positive relationships and influences of favourable organisational factors on favourable patient experiences and outcome are evident, as opposed to negative relationships. Contrary to MacDavitt et al.’s [11] conclusion that associations between organisational climate factors and patient outcomes were inconsistent, the current review shows that the increased attention in research on patient-reported variables and associations with organisational factors produced more consistent and positive results. It is also worth noting that pay-for-performance might influence the reporting of hospitals, and consequently the results of the overview of associations between the concepts in the current study. Nonetheless, exclusion of articles based in the USA (noted for the pay-for-performance system) showed a similar outlook.

Furthermore, it seems the call by MacDavitt et al. [11] to examine relationships among different patient outcome measures is being answered, looking at studies linking subjective outcomes to objective outcomes. Similarly, Flott, Darzi, Gancarczyk, and Mayer [134] maintained that there is the need to link patient experience data to other sources of data to improve healthcare practice. Thus, there is more to be done considering the increasing attention on patient-centredness, the highly dynamic health environment, and the increase in the number and complexity of illnesses.

Another notable issue is that of the measurement of patient experiences and outcomes. Most of the studies that employed secondary sources of data were in countries that had comprehensive, annual, and publicly accessible data on the study variables. In the USA, this annual data gathering from patients using the HCAHPS is particularly useful due to reforms in the health sector that link hospital performance and efficiency to hospital ratings of patient experiences and satisfaction by patients, which form the basis for hospital funding from the government. In Norway, this annual data collection is geared towards improving quality and patient experiences, and ensuring sustained hospital performance, but not necessarily as a basis for hospital funding. Beyond the Western context, countries in Asia such as Saudi Arabia have made strides towards national patient experience measurements and hospital performance indicators in recent times [7]. The question that arises then is how valid and reliable the measurement instruments are as they are used yearly. Bull, Byrnes, Hettiarachchi, and Downes [135] concluded that testing the responsiveness of PREMs is especially important when assessing changes in patient-reported variables over time. Some studies have partially or fully validated measuring instruments employed in obtaining patient-reported feedback on healthcare annually. For instance, Weidmer, Brach, Slaughter, and Hays [136] and Rothman, Park, Hays, Edwards, and Dudley [137] conducted a validation of additional items to the HCAHPS in the USA while Addo et al. [45] validated a PREM used in gathering annual patient experience data in Norway. Notwithstanding these studies, the aim to gather periodic data on patient-reported variables as well as hospital records of patients should be accompanied by more robust and periodic validation of instruments.

Moreover, despite most interventions achieving the desired effects, few of them were geared towards solving a particular problem such as enhancing patient safety and quality, improving transition and discharge experience, and reducing rates of hospital-acquired conditions in a particular hospital [36,37,112]. This relates to the practical side of developing quality-oriented care climates. By this, contextual problems that are reported by patients or recorded in hospital data can form the basis of interventions in those institutions. This would allow for context-specific problems of hospitals to be studied for developing and implementing tailor-made interventions to these problems, and consequently improving patient outcomes.

Lastly, the inconsistencies found in studies on hospital/healthcare costs and patient experiences and outcomes as well as in some studies on group comparisons need to be addressed. In relation to healthcare costs, McCaughey, Stalley, and Williams [138] studied the relationship between hospitals’ expenditure on enhancing environmental services and cleanliness, and patient experience. The study concluded that although there was no linear relationship between environmental expenses and patient experience measures, some differentiation among hospitals based on cleanliness ratings and overall spending can be drawn. Four categories of hospitals were identified: investors, high-rollers, savers, and spenders. Investors had higher levels of leadership and training of the environmental services team, and a high culture of cleanliness, among others. High-roller hospitals were seen as less efficient, had higher levels of leadership and training of the environmental services team and a high culture of cleanliness. Savers had less investment, focused leadership, and older facilities, while spenders had large investment, lower cleanliness culture, and older facilities as well. For hospitals in countries whose funding is linked to their performance, investing adequately in environmental services could improve patient experiences of healthcare, regardless of how high hospital costs are for patients or how many of the patients are considered low income. These inconsistent findings may well be due to the existing health and social systems in a country. For instance, a country whose health system is based on social welfare and subsidising health costs for all may have less significant difference in patient experience between low-income and high-income patients compared to another country where subsidised healthcare is not universal. The same argument can be made for comparisons on patient experiences and outcomes between groups, for example, between private and public hospitals.

Overall, these findings support the Donabedian framework. The overview of associations show clearly that structures and processes relate with outcomes within healthcare. As such, if management in hospitals wish to improve quality, they must pursue interventions towards healthcare quality such as team building and training. This is also buttressed by the findings under the forms of interventions which show some viability of these interventions in causing changes in outcomes. Furthermore, hospital management should develop these policies and interventions in collaboration with employees and patients to encourage commitment and successful implementation.

### 4.1. Recent Developments

This study further conducted literature searches in the same journal databases, in acknowledgment of the time that has elapsed since the last search (August 2022). This was also done to summarily compare results and boost the conclusions. The results were in line with this study’s main findings, where associations among patient experience, patient outcomes, and organisational climate factors were generally positive [7,139,140,141]. For instance, Koundakjian et al. [140] assessed the impact of a new hospital building with evidence-based design features on clinical outcomes and found that discharge from the new building was associated with a high overall hospital rating on HCAHPS. Hydoub et al. [141] also found that patient experience was associated with lower odds of 30-day hospital admission. Similar to the current study’s findings, intervention forms in more recent studies spanned from tangible to intangible ones and most of them also did not employ any theoretical underpinning.

### 4.2. The Quality Care Climate

This review showed that the Donabedian framework is the most employed in underpinning relationships among the study variables. This is not surprising as Lawson and Yazdany [123] consider it the most widely used in the healthcare sector to assess quality. Nonetheless, this framework has been seen as too simplistic. At a glance, the categories seem to stand separately and the complexities and interrelationships among them seem inadequately captured. Bjertnaes et al. [4] indicated that links are required among the categories in Donabedian’s framework. According to Nembhard et al. [25], quality improvement programmes have been pursued to improve quality-oriented climates, but their effectiveness is not known. A good starting point is an attempt towards a comprehensive framework on a quality-oriented care climate that captures the relevant aspects and complexities of healthcare, geared towards improving patient-centeredness as well as hospital efficiency and staff outcomes. Researchers have attempted to develop patients’ climate perspectives by validating a health care climate questionnaire. While these attempts have been ingenious, the studies mainly alluded to the Self-Determination Theory (SDT) by stressing patients’ autonomy and physicians support for patients to take charge of their own health [142,143,144].

**Figure 4:**
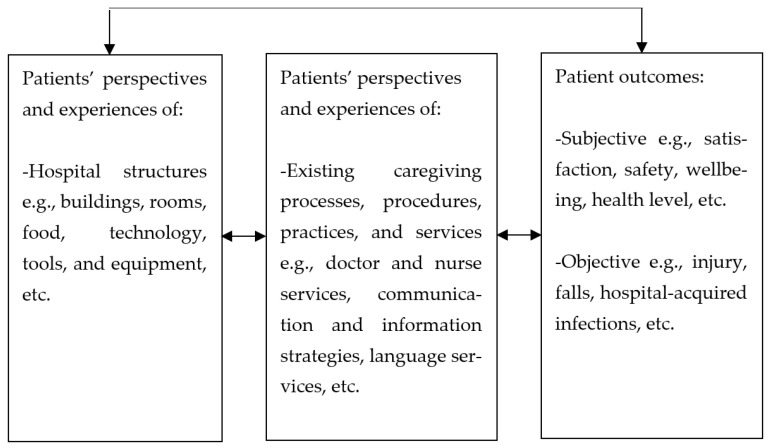
The quality care climate framework.

The SDT posits that when patients feel a greater sense of autonomy, it boosts their adherence to advice and treatment from practitioners as well as their efforts to prevent diseases [142]. If patients can be trusted to take control of their own health, can they not be equally trusted to assess the level of quality of their caregiving process as they have experienced it, and as it influences their outcomes? This study thus proposes a shift of perspectives in the organisational climate in hospitals from employees to patients, based on the trust that patients can adequately assess the tangible and intangible structures that facilitate healthcare (structure), the entire duration of caregiving and what goes into it (process), as well as their subjective and objective outcomes. The proposed care climate is therefore a measure of the level of quality in caregiving as experienced and reported by patients, and which reflects the current state of facilities and processes in hospitals. The results from this review show that the relationships among patient experiences, patient outcomes and hospital climate factors are mainly positive suggesting that positive perceptions of structures and processes will most likely relate significantly with positive outcomes giving a reliable and comprehensive indication of the existence of favourable climate factors in the hospitals from the patients’ view. This culminates into the level of the quality care climate. Figure 4 illustrates the quality care climate framework drawing on the perspective of patients and the interrelatedness of the concepts in this review.

In this framework, the major components of the quality care climate are patient perceptions and experiences, and patient outcomes. Under these, patient perspectives and experiences of tangible (buildings, food, bed, etc.) and intangible aspects (communication, information, etc.) of the hospital environment are subcomponents. Similarly, subjective and objective patient outcomes are subcomponents of patient outcomes. The quality care climate differs from other climate dimensions in the sense that while service climate and safety climate are mainly assessed from employee perspectives, the quality care climate is assessed from patients’ perspectives by the patients.

## 5. Limitations and Delimitations

However, this review is not without limitations. First, the review limited its search to quantitative studies or studies that employed mainly quantitative tools to assess associations or changes. Secondly, only peer-reviewed articles were included in the review. Also, a clear distinction was made between some related concepts, for example, between organisational climate and organisational culture, where the latter was excluded. Furthermore, the study design varied among the included articles, and this may have affected the ability to adequately synthesise. Lastly, articles from low-income and middle-income countries were somewhat underrepresented, compared to high-income countries. Regarding delimitations, the study excluded articles written in languages other than English due to the language competence of the authors. The study also included only adult inpatients and excluded studies with qualitative designs primarily. These were done to reduce variations among the studies included and improve accuracy of the synthesis. Notwithstanding these limitations, this review provides useful conclusions based on robust and systematic procedures. Future review studies could incorporate these exclusions in their searches to enhance the understanding of associations between the study variables.

Recommendations for future research: First, there is the need for further development of the quality care climate framework, both theoretically and in terms of measurement. For instance, a questionnaire can be developed and validated based on the idea of the proposed framework which captures the complexities and interrelationships among organisational factors and patient-related factors.

Secondly, the majority of the studies employed cross-sectional designs, despite a good number employing longitudinal studies and interventions. Although most of the longitudinal studies also show positive influences of favourable organisational factors on patient variables, there is still the need for more studies on concrete changes and trends in the associations of these variables.

Furthermore, more studies are needed towards linking subjective patient outcomes or experiences to objective outcomes. This is especially relevant for longitudinal studies to assess changes in patient-reported variables in relation to hospital records of outcomes that they have little control over.

Also, there should be more best practice examples in improving the experiences and outcomes of patients. As such, more quality improvement programmes should be studied by way of interventions based on reports from patients. This also means increased collaboration between researchers and hospitals to know actual issues that need intervention for improved patient experience.

It is safe to say the inconsistency in findings has shifted from the relationship between organisational climate and patient outcomes to comparisons across groups/cohorts and the relationship of healthcare costs to patient-related variables. More research is needed in drawing consistent conclusions regarding comparisons on patient experiences and outcomes across groups as well as on the relationships between healthcare costs, pricing and expenditure and patient-related variables. The existing health systems as well as the social and political systems of countries would be contributing factors to these endeavours, thus researchers should consider the contexts and locations when pursuing these research areas. These areas could therefore be extended to comparing across countries for more nuanced analysis and conclusion.

The inconsistent findings among the intervention studies also need more attention from researchers. More studies on interventions such as bedside rounds, music and massage therapy could help establish more consistent and reliable information on the effects of these interventions.

Lastly, there is the need for more studies to validate instruments that are used to gather periodic data on patient-reported variables. This is to ensure that adequate and accurate data is always gathered from patients. Moreover, if quality improvement programmes and interventions are to be based on patient-reported data, then such data must be based on consistently valid and reliable instruments.

## 6. Conclusions

This review proves substantively that the relationships between organisational climate factors and patient-related factors are more consistently positive. This study was timely and important considering both the span of years included in this review and that the last related review was in 2007. Also, these organisational factors have been seen to have significant effects on trends and levels of patient-related variables over time. Nonetheless, this review shows some dearth in quality-oriented care climate theory and practice, the need for more longitudinal studies particularly in linking subjective patient outcomes to objective patient outcomes, as well as the need for periodic validation of patient-reported instruments. Consequently, this study highlights implications for policy development from management towards improvement of the hospital climate, the experience and outcomes of patients. This study then proposes a quality care climate framework to assess patients’ perspectives of the hospital environment. Future research based on the study results is recommended.

## Figures and Tables

**Table 1:** Studies and extracted data truncated.

Author(s) and Year	Main Purpose	Main Variables	Context/Location	Theoretical/Conceptual Framework	Design	Sampling	Analytical Approach	Main Outcomes
Oppel and Mohr (2020) [13]	To investigate the mediating role of nurses’ civility towards patients between nurses’ civility climate and patient experience outcomes (overall hospital rating, willingness to return, intent to recommend).	Patient outcomes (overall hospital ratings, willingness to return, intent to recommend). Civility climate. Civility towards patients.	USA	Self-developed	Quantitative- cross-sectional survey	Secondary source	Multilevel modelling	A positive association between civility climate and civility toward patients. Direct effects of civility climate on overall hospital rating, intent to recommend, and willingness to return. An indirect effect mediated by civility toward patients.
Adams et al. (2016) [31]	To assess the relationship between patient satisfaction and perceptions of primary care physician–hospital team communication.	Patient satisfaction. Primary care physician (PCP) hospital team.	USA	NA	Mixed method, cross-sectional	Primary sources: interviews (pre- and post-discharge), surveys	Non-parametric tests (Kruskal–Wallis and chi-square)	Patients’ perception of communication was significantly associated with patient satisfaction.

## Data Availability

Supporting data on articles is shared in the Supplementary File.

## Supplementary Materials

The following supporting information can be downloaded at: https://www.mdpi.com/article/10.3390/ijerph23020268/s1. Supplementary A: Table S1: PRISMA_2020_checklist; Supplementary B: Table S2: Prisma abstracts; Supplementary C: Search string; Supplementary D: Interactive mapping of study contexts, research designs, and variables; Supplementary E: Table S3: Risk table; Supplementary F: Table S4: Studies and extracted data. References [145,146,147,148,149,150,151,152,153,154,155,156,157,158,159,160,161,162,163,164,165,166,167,168,169,170,171,172,173,174,175,176,177,178,179,180,181,182,183,184,185,186,187,188,189,190,191,192,193,194,195,196,197,198,199,200,201,202,203,204,205,206,207,208,209,210,211,212,213,214,215,216,217,218,219,220,221,222,223,224,225,226,227,228,229,230,231,232,233,234,235,236,237,238,239,240,241,242,243,244,245,246,247,248,249,250,251,252] are cited in the supplementary materials.

## References

[1] Wolf J., Niederhauser V., Marshburn D., LaVela S. (2014). Defining Patient Experience. Patient Exp. J..

[2] Lunn M.L., Ellinger A.D., Nimon K.F., Halbesleben J.R. (2021). Chief Executive Officers’ Perceptions of Collective Organizational Engagement and Patient Experience in Acute Care Hospitals. J. Patient Exp..

[3] Murray C.J., Frenk J. (2000). A framework for assessing the performance of health systems. Bull. World Health Organ..

[4] Bjertnaes O.A., Sjetne I.S., Iversen H.H. (2012). Overall patient satisfaction with hospitals: Effects of patient-reported experiences and fulfilment of expectations. BMJ Qual. Saf..

[5] Jenkinson C., Coulter A., Bruster S., Richards N., Chandola T. (2002). Patients’ experiences and satisfaction with health care: Results of a questionnaire study of specific aspects of care. Qual. Saf. Health Care.

[6] Taylor F., Halter M., Drennan V.M. (2019). Understanding patients’ satisfaction with physician assistant/associate encounters through communication experiences: A qualitative study in acute hospitals in England. BMC Health Serv. Res..

[7] Alsubahi N., Pavlova M., Alzahrani A.A., Ahmad A., Groot W. (2024). Healthcare quality from the perspective of patients in Gulf Cooperation Council countries: A systematic literature review. Healthcare.

[8] Weinberger M., Greene J.Y., Mamlin J.J. (1982). Toward a Better Understanding of the Delivery of Primary Care: The Patient, the Provider, and Their Interaction. Hum. Relat..

[9] Jones R., Jenkins F. (2007). Key Topics in Healthcare Management: Understanding the Big Picture. Key Topics in Healthcare Management: Understanding the Big Picture.

[10] Litwin G.H., Stringer R.A. (1968). Motivation and Organizational Climate.

[11] MacDavitt K., Chou S.S., Stone P.W. (2007). Organizational climate and health care outcomes. Jt. Comm. J. Qual. Patient Saf..

[12] Ogbonnaya C., Babalola M.T. (2021). A closer look at how managerial support can help improve patient experience: Insights from the UK’s National Health Service. Hum. Relat..

[13] Oppel E.M., Mohr D.C. (2020). “Paying it forward”: The link between providers’ civility climate, civility toward patients and patient experience outcomes. Health Care Manag. Rev..

[14] Oppel E.M., Mohr D.C., Benzer J.K. (2019). Let’s be civil: Elaborating the link between civility climate and hospital performance. Health Care Manag. Rev..

[15] Smirnova A., Arah O.A., Stalmeijer R.E., Lombarts K.M.J.M.H., van der Vleuten C.P.M. (2019). The Association Between Residency Learning Climate and Inpatient Care Experience in Clinical Teaching Departments in the Netherlands. Acad. Med. J. Assoc. Am. Med. Coll..

[16] Braithwaite J., Herkes J., Ludlow K., Testa L., Lamprell G. (2017). Association between organisational and workplace cultures, and patient outcomes: Systematic review. BMJ Open.

[17] Schneider B., Ehrhart M.G., Macey W.H. (2013). Organizational climate and culture. Annu. Rev. Psychol..

[18] Iversen H.H., Holmboe O., Bjertnæs O.A. (2012). The Cancer Patient Experiences Questionnaire (CPEQ): Reliability and construct validity following a national survey to assess hospital cancer care from the patient perspective. BMJ Open.

[19] Jenkinson C., Coulter A., Bruster S. (2002). The Picker Patient Experience Questionnaire: Development and validation using data from in-patient surveys in five countries. Int. J. Qual. Health Care.

[20] Pettersen K.I., Veenstra M., Guldvog B., Kolstad A. (2004). The Patient Experiences Questionnaire: Development, validity and reliability. Int. J. Qual. Health Care.

[21] Madhukar V., Sharma S. (2017). Organisational Climate: A Conceptual Perspective. Int. J. Manag. Bus..

[22] Schneider B. (1975). Organizational Climates: An Essay. Pers. Psychol..

[23] Schneider B., Bowen D.E. (1985). Employee and Customer Perceptions of Service in Banks: Replication and Extension. J. Appl. Psychol..

[24] Zohar D. (1980). Safety climate in industrial organizations: Theoretical and applied implications. J. Appl. Psychol..

[25] Nembhard I.M., Northrup V., Shaller D., Cleary P.D. (2012). Improving organizational climate for quality and quality of care: Does membership in a collaborative help?. Med. Care.

[26] Page M.J., McKenzie J.E., Bossuyt P.M., Boutron I., Hoffmann T.C., Mulrow C.D., Shamseer L., Tetzlaff J.M., Akl E.A., Brennan S.E. (2021). The PRISMA 2020 statement: An updated guideline for reporting systematic reviews. BMJ.

[27] Addo S.A., Mykletun R.J., Olsen E. Associations Among Organisational Climate Factors, Patient Experiences and Patient Outcomes Among Adult In-Patients: A Systematic Review. https://www.crd.york.ac.uk/prospero/display_record.php?ID=CRD42021291787.

[28] Moher D., Shamseer L., Clarke M., Ghersi D., Liberati A., Petticrew M., Shekelle P., Stewart L.A. (2015). Preferred reporting items for systematic review and meta-analysis protocols (PRISMA-P) 2015 statement. Syst. Rev..

[29] Thomas J., Brunton J., Graziosi S. (2010). EPPI-Reviewer 4: Software for Research Synthesis.

[30] NIH The National Institutes of Health (NIH) Quality Assessment Tool for Observational Cohort and Cross-sectional Studies. https://www.nhlbi.nih.gov/health-topics/study-quality-assessment-tools.

[31] Adams D.R., Flores A., Coltri A., Meltzer D.O., Arora V.M. (2016). A Missed Opportunity to Improve Patient Satisfaction? Patient Perceptions of Inpatient Communication with Their Primary Care Physician. Am. J. Med. Qual..

[32] Abor P.A. (2016). Healthcare governance and patients’ perception of service quality in Ghana. Int. J. Healthc. Technol. Manag..

[33] AbuDagga A., Weech-Maldonado R. (2016). Do patient, hospital, and community characteristics predict variations in overall inpatient experience scores? A multilevel analysis of hospitals in California. Health Serv. Manag. Res..

[34] Aga T.B., Ferede Y.M., Mekonen E.G. (2021). Satisfaction and associated factors towards inpatient health care services among adult patients at Pawie General Hospital, West Ethiopia. PLoS ONE.

[35] Choi N., Kim J., Kim H. (2021). The influence of patient-centeredness on patient safety perception among inpatients. PLoS ONE.

[36] Fryers M., Young L., Rowland P. (2012). Creating and sustaining a collaborative model of care. Healthc. Manag. Forum.

[37] Pottenger B.C., Davis R.O., Miller J., Allen L., Sawyer M., Pronovost P.J. (2016). Comprehensive Unit-based Safety Program (CUSP) to Improve Patient Experience: How a Hospital Enhanced Care Transitions and Discharge Processes. Qual. Manag. Health Care.

[38] Beauvais B., Richter J.P., Kim F.S., Palmer E.L., Spear B.L., Turner R.C. (2021). A reason to renovate: The association between hospital age of plant and value-based purchasing performance. Health Care Manag. Rev..

[39] McFarland D.C., Shen M.J., Parker P., Meyerson S., Holcombe R.F. (2017). Does Hospital Size Affect Patient Satisfaction?. Qual. Manag. Healthc..

[40] Roseen E.J., Cornelio-Flores O., Lemaster C., Hernandez M., Fong C., Resnick K., Wardle J., Hanser S., Saper R. Inpatient Massage Therapy Versus Music Therapy Versus Usual Care: A Mixed-Methods Feasibility Randomized Controlled Trial. https://opus.lib.uts.edu.au/handle/10453/161987.

[41] Aiken L.H., Sloane D.M., Barnes H., Cimiotti J.P., Jarrín O.F., McHugh M.D. (2018). Nurses’ and patients’ appraisals show patient safety in hospitals remains a concern. Health Aff..

[42] Andrade C.C., Lima M.L., Pereira C.R., Fornara F., Bonaiuto M. (2013). Inpatients’ and outpatients’ satisfaction: The mediating role of perceived quality of physical and social environment. Health Place.

[43] Bergholt M.D., Falstie-Jensen A.M., Brink Valentin J., Hibbert P., Braithwaite J., Johnsen S.P., Von Plessen C. (2021). Patients experience more support, information and involvement after first-time hospital accreditation: A before and after study in the Faroe Islands. Int. J. Qual. Health Care.

[44] Mazurenko O., Richter J., Kazley A.S., Ford E. (2019). Examination of the relationship between management and clinician perception of patient safety climate and patient satisfaction. Health Care Manag. Rev..

[45] Addo S.A., Mykletun R.J., Olsen E. (2021). Validation and Adjustment of the Patient Experience Questionnaire (PEQ): A Regional Hospital Study in Norway. Int. J. Environ. Res. Public Health.

[46] Asagbra O.E., Burke D., Liang H. (2019). The association between patient engagement HIT functionalities and quality of care: Does more mean better?. Int. J. Med. Inf..

[47] Belasen A.R., Tracey M.R., Belasen A.T. (2021). Demographics matter: The potentially disproportionate effect of COVID-19 on hospital ratings. Int. J. Qual. Health Care.

[48] Alhusban M.A., Abualrub R.F. (2009). Patient satisfaction with nursing care in Jordan. J. Nurs. Manag..

[49] Brener M.I., Epstein J.A., Cho J., Yeh H.C., Dudas R.A., Feldman L. (2016). Faces of all clinically engaged staff: A quality improvement project that enhances the hospitalised patient experience. Int. J. Clin. Pract..

[50] Rathert C., May D.R., Williams E.S. (2011). Beyond service quality: The mediating role of patient safety perceptions in the patient experience-satisfaction relationship. Health Care Manag. Rev..

[51] Arntson E., Dimick J.B., Nuliyalu U., Errickson J., Engler T.A., Ryan A.M. (2021). Changes in hospital-acquired conditions and mortality associated with the hospital-acquired condition reduction program. Ann. Surg..

[52] Ashana D.C., Chan V.K., Vangala S., Bell D.S. (2021). The Impact of Resident Holdover Admissions on Length of Hospital Stay and Risk of Transfer to an Intensive Care Unit. J. Patient Saf..

[53] Beaulieu N.D., Dafny L.S., Landon B.E., Dalton J.B., Kuye I., McWilliams J.M. (2020). Changes in Quality of Care after Hospital Mergers and Acquisitions. N. Engl. J. Med..

[54] Bentler S.E., Morgan R.O., Virnig B.A., Wolinsky F.D. (2014). The association of longitudinal and interpersonal continuity of care with emergency department use, hospitalization, and mortality among Medicare beneficiaries. PLoS ONE.

[55] Chen L.M., Birkmeyer J.D., Saint S., Jha A.K. (2013). Hospitalist staffing and patient satisfaction in the national Medicare population. J. Hosp. Med..

[56] Delhy R., Dor A., Pittman P. (2021). The Impact of Nursing Staff on Satisfaction Scores for U.S. Hospitals: A Production Function Approach. Med. Care Res. Rev..

[57] Bjertnaes O., Deilkås E.T., Skudal K.E., Iversen H.H., Bjerkan A.M. (2015). The association between patient-reported incidents in hospitals and estimated rates of patient harm. Int. J. Qual. Health Care.

[58] Hu L., Ding H., Liu S., Wang Z., Hu G., Liu Y. (2020). Influence of patient and hospital characteristics on inpatient satisfaction in China’s tertiary hospitals: A cross-sectional study. Health Expect..

[59] McCaughey D., McGhan G.E., Rathert C., Williams J.H., Hearld K.R. (2020). Magnetic work environments: Patient experience outcomes in Magnet versus non-Magnet hospitals. Health Care Manag. Rev..

[60] Stimpfel A.W., Sloane D.M., McHugh M.D., Aiken L.H. (2016). Hospitals Known for Nursing Excellence Associated with Better Hospital Experience for Patients. Health Serv. Res..

[61] Chatterjee P., Joynt K.E., Orav E.J., Jha A.K. (2012). Patient experience in safety-net hospitals: Implications for improving care and value-based purchasing. Arch. Intern. Med..

[62] Lappé K.L., Raaum S.E., Ciarkowski C.E., Reddy S.P., Johnson S.A. (2020). Impact of Hospitalist Team Structure on Patient-Reported Satisfaction with Physician Performance. J. Gen. Intern. Med..

[63] Chae W., Kim J., Park E.C., Jang S.I. (2021). Comparison of Patient Satisfaction in Inpatient Care Provided by Hospitalists and Nonhospitalists in South Korea. Int. J. Environ. Res. Public Health.

[64] Bachnick S., Ausserhofer D., Baernholdt M., Simon M., Match RN Study Group (2018). Patient-centered care, nurse work environment and implicit rationing of nursing care in Swiss acute care hospitals: A cross-sectional multi-center study. Int. J. Nurs. Stud..

[65] Beauvais B., Gilson G., Schwab S., Jaccaud B., Pearce T., Holmes T. (2020). Overpriced? Are Hospital Prices Associated with the Quality of Care?. Healthcare.

[66] Nembhard I.M., Yuan C.T., Shabanova V., Cleary P.D. (2015). The relationship between voice climate and patients’ experience of timely care in primary care clinics. Health Care Manag. Rev..

[67] Liu S.S., Wen Y.P., Mohan S., Bae J., Becker E.R. (2016). Addressing Medicaid Expansion from the Perspective of Patient Experience in Hospitals. Patient.

[68] Chan B., Goldman L.E., Sarkar U., Schneidermann M., Kessell E., Guzman D., Critchfield J., Kushel M. (2015). The Effect of a Care Transition Intervention on the Patient Experience of Older Multi-Lingual Adults in the Safety Net: Results of a Randomized Controlled Trial. J. Gen. Intern. Med..

[69] Rajaram R., Saadat L., Chung J., Dahlke A., Yang A.D., Odell D.D., Bilimoria K.Y. (2016). Impact of the 2011 ACGME resident duty hour reform on hospital patient experience and processes-of-care. BMJ Qual. Saf..

[70] Picone D.M., Titler M.G., Dochterman J., Shever L., Kim T., Abramowitz P., Kanak M., Qin R. (2008). Predictors of medication errors among elderly hospitalized patients. Am. J. Med. Qual..

[71] Schwendimann R., Bühler H., De Geest S., Milisen K. (2008). Characteristics of hospital inpatient falls across clinical departments. Gerontology.

[72] Fletcher G.S., Aaronson B.A., White A.A., Julka R. (2017). Effect of a Real-Time Electronic Dashboard on a Rapid Response System. J. Med. Syst..

[73] Tevis S.E., Kennedy G.D. (2015). Patient satisfaction: Does surgical volume matter?. J. Surg. Res..

[74] Gambhir H.S., Goodrick S., Dhamoon A., Kaul V. (2021). Impact of Structured and Scheduled Family Meetings on Satisfaction in Patients Admitted to Hospitalist Service. J. Patient Exp..

[75] Schwappach D.L.B., Frank O., Buschmann U., Babst R. (2013). Effects of an educational patient safety campaign on patients’ safety behaviours and adverse events. J. Eval. Clin. Pract..

[76] Wild D.M.G., Kwon N., Dutta S., Tessier-Sherman B., Woddor N., Sipsma H.L., Rizzo T., Bradley E.H. (2011). Who’s behind an HCAHPS score?. Jt. Comm. J. Qual. Patient Saf..

[77] Tevis S.E., Kennedy G.D., Kent K.C. (2015). Is There a Relationship Between Patient Satisfaction and Favorable Surgical Outcomes?. Adv. Surg..

[78] Sacks G.D., Lawson E.H., Dawes A.J., Russell M.M., Maggard-Gibbons M., Zingmond D.S., Ko C.Y. (2015). Relationship Between Hospital Performance on a Patient Satisfaction Survey and Surgical Quality. JAMA Surg..

[79] Dobrzykowski D.D., Callaway S.K., Vonderembse M.A. (2015). Examining Pathways from Innovation Orientation to Patient Satisfaction: A Relational View of Healthcare Delivery. Decis. Sci..

[80] Real K., Bell S., Williams M.V., Latham B., Talari P., Li J. (2020). Patient Perceptions and Real-Time Observations of Bedside Rounding Team Communication: The Interprofessional Teamwork Innovation Model (ITIM). Jt. Comm. J. Qual. Patient Saf..

[81] Spaulding A., Choate S., Hamadi H., Zhao M. (2018). The Impact of Hospitalists on Value-Based Purchasing Program Scores. J. Healthc. Manag..

[82] Jacobs E.A., Sadowski L.S., Rathouz P.J. (2007). The impact of an enhanced interpreter service intervention on hospital costs and patient satisfaction. J. Gen. Intern. Med..

[83] Lyu H., Wick E.C., Housman M., Freischlag J.A., Makary M.A. (2013). Patient satisfaction as a possible indicator of quality surgical care. JAMA Surg..

[84] Attebery T., Hearld L.R., Carroll N., Szychowski J., Weech-Maldonado R. (2020). Better together? An examination of the relationship between acute care hospital mergers and patient experience. J. Healthc. Manag..

[85] Stanowski A.C., Simpson K., White A. (2015). Pay for Performance: Are Hospitals Becoming More Efficient in Improving Their Patient Experience?. J. Healthc. Manag..

[86] Al-Amin M., Schiaffino M.K., Park S., Harman J. (2018). Sustained Hospital Performance on Hospital Consumer Assessment of Healthcare Providers and Systems Survey Measures: What Are the Determinants?. J. Healthc. Manag..

[87] Pérotin V., Zamora B., Reeves R., Bartlett W., Allen P. (2013). Does hospital ownership affect patient experience? An investigation into public-private sector differences in England. J. Health Econ..

[88] Charalambous M., Sisou G., Talias M. (2018). Assessment of Patients’ Satisfaction with Care Provided in Public and Private Hospitals of the Republic of Cyprus: A Comparative Study. https://www.semanticscholar.org/paper/Assessment-of-Patients-%27-Satisfaction-with-Care-in-Charalambous-Sisou/ae536a0af21ea5a0f5a7391979c11f4fa2e9a3f6.

[89] Aboshaiqah A.E., Alonazi W.B., Patalagsa J.G. (2016). Patients’ assessment of quality of care in public tertiary hospitals with and without accreditation: Comparative cross-sectional study. J. Adv. Nurs..

[90] Mabire C., Bachnick S., Ausserhofer D., Simon M., Match RN Study Group (2019). Patient readiness for hospital discharge and its relationship to discharge preparation and structural factors: A cross-sectional study. Int. J. Nurs. Stud..

[91] Sjetne I.S., Veenstra M., Stavem K. (2007). The effect of hospital size and teaching status on patient experiences with hospital care: A multilevel analysis. Med. Care.

[92] Zhu J., Dy S.M., Wenzel J., Wu A.W. (2018). Association of Magnet Status and Nurse Staffing with Improvements in Patient Experience with Hospital Care. Med. Care.

[93] Kutney-Lee A., Melendez-Torres G.J., McHugh M.D., Wall B.M. (2014). Distinct enough? A national examination of Catholic hospital affiliation and patient perceptions of care. Health Care Manag. Rev..

[94] Kang R., Hasnain-Wynia R. (2013). Hospital commitment to community orientation and its association with quality of care and patient experience. J. Healthc. Manag..

[95] Kang Y.S., Tzeng H.M., Zhang T. (2020). Rural Disparities in Hospital Patient Satisfaction: Multilevel Analysis of the Massachusetts AHA, SID, and HCAHPS Data. J. Patient Exp..

[96] Nemati R., Bahreini M., Pouladi S., Mirzaei K., Mehboodi F. (2020). Hospital service quality based on HEALTHQUAL model and trusting nurses at Iranian university and non-university hospitals: A comparative study. BMC Nurs..

[97] Wray C.M., Flores A., Padula W.V., Prochaska M.T., Meltzer D.O., Arora V.M. (2016). Measuring patient experiences on hospitalist and teaching services: Patient responses to a 30-day postdischarge questionnaire. J. Hosp. Med..

[98] DiLeo R., Borkowski N., O’Connor S.J., Datti P., Weech-Maldonado R. (2020). The Relationship Between “Leader in LGBT Healthcare Equality” Designation and Hospitals’ Patient Experience Scores. J. Healthc. Manag..

[99] Khalil V. (2020). Evaluating the impact of various medication safety risk reduction strategies on medication errors in an Australian Health Service. Int. J. Clin. Pharm..

[100] Kline T.J.B., Baylis B.W., Chatur F., Morrison S.A., White D.E., Flin R.H., Ghali W.A. (2007). Patient satisfaction: Evaluating the success of hospital ward redesign. J. Healthc. Qual..

[101] Siddiqui Z.K., Zuccarelli R., Durkin N., Wu A.W., Brotman D.J. (2015). Changes in patient satisfaction related to hospital renovation: Experience with a new clinical building. J. Hosp. Med..

[102] Burritt J.E., Wallace P., Steckel C., Hunter A. (2007). Achieving quality and fiscal outcomes in patient care: The clinical mentor care delivery model. J. Nurs. Adm..

[103] Grimes T.C., Deasy E., Allen A., O’BYrne J., Delaney T., Barragry J., Breslin N., Moloney E., Wall C. (2014). Collaborative pharmaceutical care in an Irish hospital: Uncontrolled before-after study. BMJ Qual. Saf..

[104] Opper K., Beiler J., Yakusheva O., Weiss M. (2019). Effects of Implementing a Health Team Communication Redesign on Hospital Readmissions Within 30 Days. Worldviews Evid. Based Nurs..

[105] Hahn-Goldberg S., Okrainec K., Damba C., Huynh T., Lau D., Maxwell J., McGuire R., Yang L., Abrams H.B. (2016). Implementing Patient-Oriented Discharge Summaries (PODS): A Multisite Pilot Across Early Adopter Hospitals. Healthc. Q..

[106] Manville M., Klein M.C., Bainbridge L. (2014). Improved outcomes for elderly patients who received care on a transitional care unit. Can. Fam. Physician.

[107] Oh E.G., Kim J.H., Lee H.J. (2019). Effects of a safe transition programme for discharged patients with high unmet needs. J. Clin. Nurs..

[108] Dunn A.S., Reyna M., Radbill B., Parides M., Colgan C., Osio T., Benson A., Brown N., Cambe J., Zwerling M. (2017). The Impact of Bedside Interdisciplinary Rounds on Length of Stay and Complications. J. Hosp. Med..

[109] Monash B., Najafi N., Mourad M., Rajkomar A., Ranji S.R., Fang M.C., Glass M., Milev D., Ding Y., Shen A. (2017). Standardized Attending Rounds to Improve the Patient Experience: A Pragmatic Cluster Randomized Controlled Trial. J. Hosp. Med..

[110] O’Leary K.J., Killarney A., Hansen L.O., Jones S., Malladi M., Marks K., Shah H.M. (2016). Effect of patient-centred bedside rounds on hospitalised patients’ decision control, activation and satisfaction with care. BMJ Qual. Saf..

[111] Hardin S.R., Dienemann J., Rudisill P., Mills K.K. (2013). Inpatient fall prevention: Use of in-room Webcams. J. Patient Saf..

[112] Hyman D., Neiman J., Rannie M., Allen R., Swietlik M., Balzer A. (2017). Innovative Use of the Electronic Health Record to Support Harm Reduction Efforts. Pediatrics.

[113] Eamranond P.P., Bhukhen A., DiPalma D., Kunuakaphun S., Burke T., Rodis J., Grey M. (2020). Interprofessional, multitiered daily rounding management in a high-acuity hospital. Int. J. Health Care Qual. Assur..

[114] Wilson C., Whiteman K., Stephens K., Swanson-Biearman B., LaBarba J. (2017). Improving the Patient’s Experience with a Multimodal Quiet-at-Night Initiative. J. Nurs. Care Qual..

[115] Yinger O.S., Standley J.M. (2011). The effects of medical music therapy on patient satisfaction: As measured by the Press Ganey Inpatient Survey. Music. Ther. Perspect..

[116] Silvera-Tawil D., Pocock C., Bradford D., Donnell A., Freyne J., Harrap K., Brinkmann S. (2021). Enabling Nurse-Patient Communication with a Mobile App: Controlled Pretest-Posttest Study with Nurses and Non-English-Speaking Patients. JMIR Nurs..

[117] Rotman-Pikielny P., Rabin B., Amoyal S., Mushkat Y., Zissin R., Levy Y. (2007). Participation of family members in ward rounds: Attitude of medical staff, patients and relatives. Patient Educ. Couns..

[118] O’Leary K.J., Lindquist L.A., Colone M.A., Haviley C., Thompson J.A., Baker D.W. (2008). Effect of a hospitalist-care coordinator team on a nonteaching hospitalist service. J. Hosp. Med..

[119] Stokes J., Shah V., Goldzahl L., Kristensen S.R., Sutton M. (2021). Does prevention-focused integration lead to the triple aim? An evaluation of two new care models in England. J. Health Serv. Res. Policy.

[120] Hanskamp-Sebregts M., Zegers M., Westert G.P., Boeijen W., Teerenstra S., van Gurp P.J., Wollersheim H. (2019). Effects of patient safety auditing in hospital care: Results of a mixed-method evaluation (part 1). Int. J. Qual. Health Care.

[121] Timmermans M.J.C., van Vught A.J.A.H., Peters Y.A.S., Meermans G., Peute J.G.M., Postma C.T., Smit P.C., Verdaasdonk E., Reilingh T.S.D.V., Wensing M. (2017). The impact of the implementation of physician assistants in inpatient care: A multicenter matched-controlled study. PLoS ONE.

[122] Donabedian A. (1988). The quality of care. How can it be assessed?. JAMA.

[123] Lawson E.F., Yazdany J. (2012). Healthcare quality in systemic lupus erythematosus: Using Donabedian’s conceptual framework to understand what we know. Int. J. Clin. Rheumatol..

[124] Andaleeb S.S., Siddiqui N., Khandakar S. (2007). Patient satisfaction with health services in Bangladesh. Health Policy Plan..

[125] Chaabouni S., Abednnadher C. (2014). The relationship between patient satisfaction and service quality: A study of hospitals in Tunisia. Indian J. Health Wellbeing.

[126] Lee M.A., Yom Y.H. (2007). A comparative study of patients’ and nurses’ perceptions of the quality of nursing services, satisfaction and intent to revisit the hospital: A questionnaire survey. Int. J. Nurs. Stud..

[127] Diana M.L., Zhang Y., Yeager V.A., Stoecker C., Counts C.R. (2019). The impact of accountable care organization participation on hospital patient experience. Health Care Manag. Rev..

[128] Bacon C.T., Hughes L.C., Mark B.A. (2009). Organizational influences on patient perceptions of symptom management. Res. Nurs. Health.

[129] Kaiser F., Schmid A., Schlüchtermann J. (2020). Physician-leaders and hospital performance revisited. Soc. Sci. Med..

[130] Liang C., Gu D., Tao F., Jain H.K., Zhao Y., Ding B. (2017). Influence of mechanism of patient-accessible hospital information system implementation on doctor–patient relationships: A service fairness perspective. Inf. Manag..

[131] Shin N., Park J. (2018). The Effect of Intentional Nursing Rounds Based on the Care Model on Patients’ Perceived Nursing Quality and their Satisfaction with Nursing Services. Asian Nurs. Res..

[132] Chakraborty S., Church E.M. (2020). Social media hospital ratings and HCAHPS survey scores. J. Health Organ. Manag..

[133] Mahmood F.J., Tayib A.Y. (2020). The Role of Patients’ Psychological Comfort in Optimizing Indoor Healing Environments: A Case Study of the Indoor Environments of Recently Built Hospitals in Sulaimani City, Kurdistan, Iraq. Health Environ. Res. Des. J..

[134] Flott K., Darzi A., Gancarczyk S., Mayer E. (2018). Improving the Usefulness and Use of Patient Survey Programs: National Health Service Interview Study. J. Med. Internet Res..

[135] Bull C., Byrnes J., Hettiarachchi R., Downes M. (2019). A systematic review of the validity and reliability of patient-reported experience measures. Health Serv. Res..

[136] Weidmer B.A., Brach C., Slaughter M.E., Hays R.D. (2012). Development of items to assess patients’ health literacy experiences at hospitals for the Consumer Assessment of Healthcare Providers and Systems (CAHPS) Hospital Survey. Med. Care.

[137] Rothman A.A., Park H., Hays R.D., Edwards C.A., Dudley R.A. Can Additional Patient Experience Items Improve the Reliability of and Add New Domains to the CAHPS Hospital Survey?. https://www.rand.org/pubs/external_publications/EP20081205.html.

[138] McCaughey D., Stalley S., Williams E. (2013). Examining the effect of EVS spending on HCAHPS scores: A value optimization matrix for expense management. J. Healthc. Manag..

[139] Hussain A., Ruowei W., Xia X., Jameel A., Chunhong S., Ahmad S. (2025). A cross-sectional study on the impact of administrative procedures and medical staff services on patient satisfaction with trust in public sector hospitals: The moderating role of e-health knowledge. BMC Health Serv. Res..

[140] Koundakjian D.C., Tompkins B.J., Repp A.B. (2023). Evaluation of a New Hospital Building’s Impact on Clinical Outcomes and Consumer Experience in Medical Inpatients. Am. J. Med. Qual..

[141] Hydoub Y.M., Fischer K.M., Hanson K.T., Coons T.J., Wilshusen L.L., Vista T.L., Colbenson G.A., Burton M.C., Habermann E.B., Dugani S.B. (2023). Multisite analysis of patient experience scores and risk of hospital admission: A retrospective cohort study. Hosp. Pract..

[142] Czajkowska Z., Wang H., Hall N.C., Sewitch M., Körner A. (2017). Validation of the English and French versions of the Brief Health Care Climate Questionnaire. Health Psychol. Open.

[143] Heissel A., Pietrek A., Rapp M.A., Heinzel S., Williams G. (2020). Perceived Health Care Climate of Older People Attending an Exercise Program: Validation of the German Short Version of the Health Care Climate Questionnaire. J. Aging Phys. Act..

[144] Schmidt K., Gensichen J., Petersen J.J., Szecsenyi J., Walther M., Williams G., Freund T. (2012). Autonomy support in primary care—validation of the German version of the Health Care Climate Questionnaire. J. Clin. Epidemiol..

[145] Barnali B., Basu R.P. (2020). A Study on the Experience of Patients regarding the Quality of Healthcare services provided in the Alipurduar District of India. J. Healthc. Qual. Res..

[146] Aiken L.H., Sloane D.M., Ball J., Bruyneel L., Rafferty A.M., Griffiths P. (2018). Patient satisfaction with hospital care and nurses in England: An observational study. BMJ Open.

[147] Al-Refaie A. (2011). A Structural Model to Investigate Factors Affect Patient Satisfaction and Revisit Intention in Jordanian Hospitals. IJALR.

[148] Aoki T., Yamamoto Y., Nakata T. (2020). Translation, adaptation and validation of the Hospital Consumer Assessment of Healthcare Providers and Systems (HCAHPS) for use in Japan: A multicentre cross-sectional study. BMJ Open.

[149] Arab M., Tabatabaei S.G., Rashidian A., Forushani A.R., Zarei E. (2012). The Effect of Service Quality on Patient loyalty: A Study of Private Hospitals in Tehran, Iran. Iran. J. Public Health.

[150] Asmaryadi A., Pasinringi S.A., Thamrin Y., Muis M. (2020). Influence of Patient Experience and Hospital Image on Patient Loyalty in Meloy Public Hospital of Sangatta, East Kutai Regency. Open Access Maced. J. Med. Sci..

[151] Auerbach A.D., Kripalani S., Vasilevskis E.E., Sehgal N., Lindenauer P.K., Metlay J.P., Fletcher G., Ruhnke G.W., Flanders S.A., Kim C. (2016). Preventability and Causes of Readmissions in a National Cohort of General Medicine Patients. JAMA Intern Med..

[152] Baek O.J., Shin S.H. (2021). The Moderating Effect of Patient Safety Knowledge in the Relationship between Patient Experience and Patient Safety Perception for Patients in Primary Care Institutions. Korean J. Adult Nurs..

[153] Bilimoria K.Y., Chung J.W., Minami C.A., Sohn M.-W., Pavey E.S., Holl J.L., Mello M.M. (2017). Relationship Between State Malpractice Environment and Quality of Health Care in the United States. Jt. Comm. J. Qual. Patient Saf..

[154] Bjertnaes O., Skudal K.E., Iversen H.H., Lindahl A.K. (2013). The Patient-Reported Incident in Hospital Instrument (PRIH-I): Assessments of data quality, test–retest reliability and hospital-level reliability. BMJ Qual. Saf..

[155] Blay N., Roche M., Duffield C., Xu X. (2017). Intrahospital transfers and adverse patient outcomes: An analysis of administrative health data. J. Clin. Nurs..

[156] Bleich S.N., Özaltin E., Murray C.J. (2009). How does satisfaction with the health-care system relate to patient experience?. Bull. World Health Organ..

[157] Burston S., Chaboyer W., Gillespie B., Carroll R. (2014). The effect of a transforming care initiative on patient outcomes in acute surgical units: A time series study. J. Adv. Nurs..

[158] Carter J.C., Silverman F.N. (2016). Using HCAHPS data to improve hospital care quality. TQM J..

[159] Chakraborty S., Church E.M. (2021). Patient hospital experience and satisfaction on social media. Int. J. Qual. Serv. Sci..

[160] Chen X., Zhang Y., Qin W., Yu Z., Yu J., Lin Y., Li X., Zheng Z., Wang Y. (2022). How does overall hospital satisfaction relate to patient experience with nursing care? a cross-sectional study in China. BMJ Open.

[161] Clark P.A., Leddy K., Drain M., Kaldenberg D. (2007). State Nursing Shortages and Patient Satisfaction. J. Nurs. Care Qual..

[162] Menendez M.E., Ring D. (2015). Do Hospital-Acquired Condition Scores Correlate with Patients’ Perspectives of Care?. Qual. Manag. Healthc..

[163] Doubova S.V., Infante-Castañeda C., Roder-DeWan S., Pérez-Cuevas R. (2019). User experience and satisfaction with specialty consultations and surgical care in secondary and tertiary level hospitals in Mexico. BMC Health Serv. Res..

[164] Durant D.J. (2021). Can patient-reported room cleanliness measures predict hospital-acquired C. difficile infection? A study of acute care facilities in New York state. Am. J. Infect. Control..

[165] Edvardsson D., Watt E., Pearce F. (2016). Patient experiences of caring and person-centredness are associated with perceived nursing care quality. J. Adv. Nurs..

[166] Lee S., Wang W., Washburn D.J., Shi H., Yu Y., Du Y., Zhang H., Maddock J.E. (2018). Effect of the treatment-before-deposit policy on trust in physicians and perceived service quality among patients in 12 hospitals in China. Int. J. Health Plan. Manag..

[167] Ernawaty, Supriyanto S., Krisbianto, Visianti (2020). The effect of hospital service quality on inpatient satisfaction in piru hospital. J. Health Transl. Med..

[168] Craig A.R., Otani K., Herrmann P.A. (2015). Evaluating the Influence of Perceived Pain Control on Patient Satisfaction in a Hospital Setting. Hosp. Top..

[169] Fatima T., Alam Malik S., Shabbir A. (2018). Hospital healthcare service quality, patient satisfaction and loyalty: An Investigation in the Context of Private Healthcare Systems. Int. J. Qual. Reliab. Manag..

[170] Figueroa J.F., Feyman Y., Zhou X., Maddox K.J. (2018). Hospital-level care coordination strategies associated with better patient experience. BMJ Qual. Saf..

[171] Fiorentini G., Robone S., Verzulli R. (2017). How do hospital-specialty characteristics influence health system responsiveness? An empirical evaluation of in-patient care in the Italian region of Emilia-Romagna. Health Econ..

[172] Gavurova B., Dvorsky J., Popesko B. (2021). Patient Satisfaction Determinants of Inpatient Healthcare. Int. J. Environ. Res. Public Health.

[173] Grifka A., Dorris J., Marshall-Aiyelawo K., Gliner M., Frazier C. (2022). Patient Experience and Hospital Environment Measures at Military Treatment Facilities. J. Healthc. Manag..

[174] Grøndahl V.A., Kirchhoff J.W., Andersen K.L., Sørby L.A., Andreassen H.M., Skaug E.-A., Roos A.K., Tvete L.S., Helgesen A.K. (2018). Health care quality from the patients’ perspective: A comparative study between an old and a new, high-tech hospital. J. Multidiscip. Healthc..

[175] Chen H., Li M., Wang J., Xue C., Ding T., Nong X., Liu Y., Zhang L. (2016). Factors influencing inpatients’ satisfaction with hospitalization service in public hospitals in Shanghai, People’s Republic of China. Patient Prefer. Adherence.

[176] Hartwell H.J., Shepherd P.A., Edwards J.S., Johns N. (2016). What do patients value in the hospital meal experience?. Appetite.

[177] Hod R., Maimon O., Zimlichman E. (2020). Does Care Transition Matter? Exploring the Newly Published HCAHPS Measure. Am. J. Med Qual..

[178] Holzer B.M., Minder C.E. (2011). A simple approach to fairer hospital benchmarking using patient experience data. Int. J. Qual. Health Care.

[179] Sturdivant T., Herrin K., Reynolds M., Mestas L. (2020). Improving patient satisfaction through a nurse leader-physician bedside rounding protocol: A pilot project. Nurs. Econ..

[180] Isaac T., Zaslavsky A.M., Cleary P.D., Landon B.E. (2010). The Relationship between Patients’ Perception of Care and Measures of Hospital Quality and Safety. Health Serv. Res..

[181] Jarvis B.M.-H., Johnson T., Butler P.M., O’shaughnessy K.M.-H., Fullam F.M., Tran L.M., Gupta R.M. (2013). Assessing the Impact of Electronic Health Records as an Enabler of Hospital Quality and Patient Satisfaction. Acad. Med..

[182] Jha A.K., Orav E.J., Zheng J., Epstein A.M. (2008). Patients’ Perception of Hospital Care in the United States. N. Engl. J. Med..

[183] Kaya S., Guven G.S., Aydan S., Kar A., Teleş M., Yıldız A., Koca G.Ş., Kartal N., Korku C., Ürek D. (2018). Patients’ readiness for discharge: Predictors and effects on unplanned readmissions, emergency department visits and death. J. Nurs. Manag..

[184] Keller A.C., Bergman M.M., Heinzmann C., Todorov A., Weber H., Heberer M. (2013). The relationship between hospital patients’ ratings of quality of care and communication. Int. J. Qual. Health Care.

[185] Kilpatrick K., Tchouaket E., Fernandez N., Jabbour M., Dubois C.-A., Paquette L., Landry V., Gauthier N., Beaulieu M.-D. (2021). Patient and family views of team functioning in primary healthcare teams with nurse practitioners: A survey of patient-reported experience and outcomes. BMC Fam. Pr..

[186] King P.K., Martin S.J., Betka E.M. (2016). Patient Awareness and Expectations of Pharmacist Services During Hospital Stay. J. Pharm. Pr..

[187] Krol M.W., De Boer D., Sixma H., Van Der Hoek L., Rademakers J.J.D.J.M., Delnoij D.M. (2014). Patient experiences of inpatient hospital care: A department matter and a hospital matter. Int. J. Qual. Health Care.

[188] Lawal B.J., Agbla S.C., Bola-Lawal Q.N., O Afolabi M., Ihaji E. (2018). Patients’ Satisfaction with Care from Nigerian Federal Capital Territory’s Public Secondary Hospitals: A Cross-Sectional Study. J. Patient Exp..

[189] Lehrman W.G., Elliott M.N., Goldstein E., Beckett M.K., Klein D.J., Giordano L.A. (2009). Characteristics of Hospitals Demonstrating Superior Performance in Patient Experience and Clinical Process Measures of Care. Med Care Res. Rev..

[190] Tzeng H.-M.P., Hu H.M.P., Yin C.-Y.M., Johnson D.M. (2011). Link Between Patients’ Perceptions of Their Acute Care Hospital Experience and Institutions’ Injurious Fall Rates. J. Nurs. Care Qual..

[191] Linnander E., McNatt Z., Sipsma H., Tatek D., Abebe Y., Endeshaw A., Bradley E.H. (2016). Use of a national collaborative to improve hospital quality in a low-income setting. Int. Health.

[192] Liu X., Zheng J., Liu K., Baggs J.G., Liu J., Wu Y., You L. (2019). Associations of nurse education level and nurse staffing with patient experiences of hospital care: A cross sectional study in China. Res. Nurs. Health.

[193] Lu C.Y., Roughead E. (2011). Determinants of patient-reported medication errors: A comparison among seven countries. Int. J. Clin. Pr..

[194] Mache S., Vitzthum K., Klapp B.F., Groneberg D.A. (2012). Improving quality of medical treatment and care: Are surgeons’ working conditions and job satisfaction associated to patient satisfaction?. Langenbeck’s Arch. Surg..

[195] Martsolf G.R., Gibson T.B., Benevent R., Jiang H.J., Stocks C., Ehrlich E.D., Kandrack R., Auerbach D.I. (2016). An Examination of Hospital Nurse Staffing and Patient Experience with Care: Differences between Cross-Sectional and Longitudinal Estimates. Health Serv. Res..

[196] McLean C., Griffiths P., Eguiagaray I.M., Pickering R.M., Bridges J. (2017). Reliability, feasibility, and validity of the quality of interactions schedule (QuIS) in acute hospital care: An observational study. BMC Health Serv. Res..

[197] Merel S.E., McKinney C.M., Ufkes P., Kwan A.C., White A.A. (2016). Sitting at patients’ bedsides may improve patients’ perceptions of physician communication skills. J. Hosp. Med..

[198] Min R., Li L., Zi C., Fang P., Wang B., Tang C. (2019). Evaluation of patient experience in county-level public hospitals in China: A multicentred, cross-sectional study. BMJ Open.

[199] Mira J.J., Lorenzo S., Navarro I. (2013). Hospital Reputation and Perceptions of Patient Safety. Med. Princ. Pr..

[200] Mitchell J.P. (2016). Electronic Healthcare’s Relationship with Patient Satisfaction and Communication. J. Healthc. Qual..

[201] Moore S.D., Wright K.B., Bernard D.R. (2009). Influences on Health Delivery System Satisfaction: A Partial Test of the Ecological Model. Health Commun..

[202] Moreira A.C., Silva P.M. (2015). The trust-commitment challenge in service quality-loyalty relationships. Int. J. Health Care Qual. Assur..

[203] Murante A.M., Vainieri M., Rojas D., Nuti S. (2014). Does feedback influence patient—Professional communication? Empirical evidence from Italy. Health Policy.

[204] Nahlah A., Palutturi S., Abadi M.Y. (2019). Factors Related to the Satisfaction of Patients in Pelamonia Hospital. Indian J. Public Health Res. Dev..

[205] Ng J., Popova S., Yau M., Sulman J. (2007). Do Culturally Sensitive Services for Chinese In-Patients Make a Difference?. Soc. Work Health Care.

[206] O’hAnlon C.E., Kranz A.M., DeYoreo M., Mahmud A., Damberg C.L., Timbie J. (2019). Access, Quality, And Financial Performance of Rural Hospitals Following Health System Affiliation. Health Aff..

[207] Olds D.M., Aiken L.H., Cimiotti J.P., Lake E.T. (2017). Association of nurse work environment and safety climate on patient mortality: A cross-sectional study. Int. J. Nurs. Stud..

[208] Omer K., Cockcroft A., Andersson N. (2011). Impact of a hospital improvement initiative in Bangladesh on patient experiences and satisfaction with services: Two cross-sectional studies. BMC Health Serv. Res..

[209] Ommen O., Wirtz M., Janssen C., Neumann M., Driller E., Ernstmann N., Loeffert S., Pfaff H. (2009). Psychometric evaluation of an instrument to assess patient-reported ‘psychosocial care by physicians’: A structural equation modeling approach. Int. J. Qual. Health Care.

[210] Olorunfemi O., Osunde N.R., Chukwuka L., Oyewole O.M., Olawale M.O. (2020). Quality of nursing care assessment in the context of coronavirus disease (COVID-19) pandemic in the University of Benin teaching hospital, Benin-City, Nigeria: Patients’ perspectives. Niger. J. Basic Clin. Sci..

[211] Otani K., Waterman B., Faulkner K.M., Boslaugh S., Dunagan C.W. (2010). How Patient Reactions to Hospital Care Attributes Affect the Evaluation of Overall Quality of Care, Willingness to Recommend, and Willingness to Return. J. Healthc. Manag..

[212] Otani K., A Herrmann P., Kurz R.S. (2011). Improving patient satisfaction in hospital care settings. Health Serv. Manag. Res..

[213] Padma P., Rajendran C., Lokachari P.S. (2010). Service quality and its impact on customer satisfaction in Indian hospitals. Benchmarking Int. J..

[214] Panchapakesan P., Sai L.P., Rajendran C. (2015). Customer Satisfaction in Indian Hospitals: Moderators and Mediators. Qual. Manag. J..

[215] Richter J.P., Muhlestein D.B. (2017). Patient experience and hospital profitability: Is there a link?. Health Care Manag. Rev..

[216] Jubelt L.E., Graham J., Maeng D.D., Li H., Epstein A.J., Metlay J.P. (2014). Patient Ratings of Case Managers in a Medical Home: Associations With Patient Satisfaction and Health Care Utilization. Ann. Intern. Med..

[217] Stein S.M., Day M., Karia R., Hutzler L., Bosco J.A. (2014). Patients’ Perceptions of Care Are Associated with Quality of Hospital Care. Am. J. Med Qual..

[218] Perera S., Dabney B.W. (2020). Case management service quality and patient-centered care. J. Health Organ. Manag..

[219] Pérez E., Dzubay D.P. (2021). A scheduling-based methodology for improving patient perceptions of quality of care in intensive care units. Health Care Manag. Sci..

[220] Peršolja M. (2018). The effect of nurse staffing patterns on patient satisfaction and needs: A cross-sectional study. J. Nurs. Manag..

[221] Lei P., Jolibert A. (2012). A three-model comparison of the relationship between quality, satisfaction and loyalty: An empirical study of the Chinese healthcare system. BMC Health Serv. Res..

[222] Ahmad I., Nawaz A., Khan S., Khan H., Rashid M.A., Khan M.H. (2011). Predictors of patient satisfaction. Gomal J. Med. Sci..

[223] Purvis T.E., Crowe T.Y., Wright S.M., Teague P. (2017). Patient Appreciation of Student Chaplain Visits During Their Hospitalization. J. Relig. Health.

[224] Ramirez J., Singh J., Williams A.A. (2016). Patient Satisfaction with Bedside Teaching Rounds Compared with Nonbedside Rounds. South. Med J..

[225] Ramsay P., Huby G., Merriweather J., Salisbury L., Rattray J., Griffith D., Walsh T. (2016). Patient and carer experience of hospital-based rehabilitation from intensive care to hospital discharge: Mixed methods process evaluation of the RECOVER randomised clinical trial. BMJ Open.

[226] Saman D.M., Kavanagh K.T., Johnson B., Lutfiyya M.N. (2013). Can Inpatient Hospital Experiences Predict Central Line-Associated Bloodstream Infections?. PLoS ONE.

[227] Scheffer C., Valk-Draad M.P., Tauschel D., Büssing A., Humbroich K., Längler A., Zuzak T., Köster W., Edelhäuser F., Lutz G. (2018). Students with an autonomous role in hospital care—Patients perceptions. Med Teach..

[228] Schnipper J.L., Nieva H.R., Mallouk M., Mixon A., Rennke S., Chu E., Mueller S., Smith G.G.R., Williams M.V., Wetterneck T.B. (2021). Effects of a refined evidence-based toolkit and mentored implementation on medication reconciliation at 18 hospitals: Results of the MARQUIS2 study. BMJ Qual. Saf..

[229] Shahijan M.K., Rezaei S., Preece C.N., Ismail W.K.W. (2015). International Medical Travelers’ Behavioral Intention: An Empirical Study in Iran. J. Travel Tour. Mark..

[230] Shen H.-C., Chiu H.-T., Lee P.-H., Hu Y.-C., Chang W.-Y. (2010). Hospital environment, nurse-physician relationships and quality of care: Questionnaire survey. J. Adv. Nurs..

[231] Silva A.G.G., Ferreira P.L., Daniel F.B. (2018). Portuguese university hospital patient satisfaction and service quality. Int. J. Health Care Qual. Assur..

[232] Steffen P., Ommen O., Pfaff H. (2009). Reduced patient demands in hospitals and their determinants. Int. J. Public Health.

[233] Swankoski K.E., Peikes D.N., Morrison N., Holland J.J., Duda N., Clusen N.A., Day T.J., Brown R.S. (2018). Patient Experience During a Large Primary Care Practice Transformation Initiative. Am. J. Manag. Care.

[234] Swiger P.A., Loan L.A., Raju D., Breckenridge-Sproat S.T., Miltner R.S., Patrician P.A. (2018). Relationships between Army nursing practice environments and patient outcomes. Res. Nurs. Health.

[235] Brooks Carthon J.M., Hatfield L.P., Brom H.P., Houton M.B., Kelly-Hellyer E.M., Schlak A.B., Aiken L.H.P. (2020). System-Level Improvements in Work Environments Lead to Lower Nurse Burnout and Higher Patient Satisfaction. J. Nurs. Care Qual..

[236] Szyca R., Rosiek A., Nowakowska U., Leksowski K. (2012). Analysis of Factors Influencing Patient Satisfaction with Hospital Treatment at the Surgical Department. Pol. J. Surg..

[237] Al-Amin M., Makarem S.C. (2016). The Effects of Hospital-Level Factors on Patients’ Ratings of Physician Communication. J. Healthc. Manag..

[238] Van Gent J.-M., Davis K.L., Henry N., Zander A.L., A Kuettel M., Edson T., Nelson T.J., Tadlock M.D. (2018). The Initial Impact of Tele-Critical Care on the Surgical Services of a Community Military Hospital. Mil. Med..

[239] Tillmann B.W., Klingel M.L., McLeod S.L., Anderson S., Haddara W., Parry N.G. (2018). The impact of delayed critical care outreach team activation on in-hospital mortality and other patient outcomes: A historical cohort study. Can. J. Anaesth..

[240] Trzeciak S., Gaughan J.P., Bosire J., Mazzarelli A.J. (2016). Association Between Medicare Summary Star Ratings for Patient Experience and Clinical Outcomes in US Hospitals. J. Patient Exp..

[241] Tzeng H., Yin C. (2008). Heights of occupied patient beds: A possible risk factor for inpatient falls. J. Clin. Nurs..

[242] Mullen D., Chessnoe J., Mettner J., Holte A., Getchell C., Bissen J. (2018). Upgrading the Quality of Hospital Linens: Effects on Patient and Staff Satisfaction. Medsurg Nursing.

[243] Zhao M., Haley D.R.P., Spaulding A.P., Balogh H.A.M. (2015). Value-Based Purchasing, Efficiency, and Hospital Performance. Health Care Manag..

[244] Vidán M.T., Sánchez E., Alonso M., Montero B., Ortiz J., Serra J.A. (2009). An Intervention Integrated into Daily Clinical Practice Reduces the Incidence of Delirium During Hospitalization in Elderly Patients. J. Am. Geriatr. Soc..

[245] Vink S., Fareed N., MacEwan S.R., McAlearney A.S. (2019). An Exploration of the Association between Inpatient Access to Tablets and Patient Satisfaction with Hospital Care. Perspect. Health Inf. Manag..

[246] Wang E., Arnold S., Jones S., Zhang Y., Volpicelli F., Weisstuch J., Horwitz L., Rudy B. (2022). Quality and Safety Outcomes of a Hospital Merger Following a Full Integration at a Safety Net Hospital. JAMA Netw. Open.

[247] Wong E.L., Leung M.C., Cheung A.W., Yam C.H., Yeoh E., Griffiths S. (2011). A population-based survey using PPE-15: Relationship of care aspects to patient satisfaction in Hong Kong. Int. J. Qual. Health Care.

[248] You L.-M., Aiken L.H., Sloane D.M., Liu K., He G.-P., Hu Y., Jiang X.-L., Li X.-H., Li X.-M., Liu H.-P. (2013). Hospital nursing, care quality, and patient satisfaction: Cross-sectional surveys of nurses and patients in hospitals in China and Europe. Int. J. Nurs. Stud..

[249] Zarei E., Daneshkohan A., Khabiri R., Arab M. (2014). The Effect of Hospital Service Quality on Patient’s Trust. Iran. Red Crescent Med. J..

[250] Zhi M., He Z., Ji J., Lian J., Guo R., Sun J., Liu Y. (2022). Patient satisfaction with non-clinical nursing care provided by the nursing assistant under different management models in Chinese public tertiary hospital. Appl. Nurs. Res..

[251] Zhu Y., Li Y., Wu M., Fu H. (2022). How do Chinese people perceive their healthcare system? Trends and determinants of public satisfaction and perceived fairness, 2006–2019. BMC Health Serv. Res..

[252] Zineldin M. (2015). Determinants of patient safety, satisfaction and trust: With focus on physicians-nurses performance. Clin. Gov. Int. J..

