# Supplementary material for: Towards a Quality Care Climate Perspective: A Systematic Review of Associations Among Patient Experience, Patient Outcomes, and Organisational Climate Factors in Hospitals
Source: Int J Environ Res Public Health. 2026 Feb 20;23(2):268. doi: 10.3390/ijerph23020268 (PMC12940218; doi:10.3390/ijerph23020268)
Supplement: Supplementary file 1 [file ijerph-23-00268-s001.zip › Supplementary C. Search string.pdf]

Supplementary C- Search string

(patient* experience*) OR (patient* perspective* (synonyms for the topic)) OR (patient* view* (synonyms for the topic)) OR (patient* opinion*) (synonyms for the topic)) AND (patient* outcome*) OR (patient* satisfy* (synonyms for the topic)) OR (patient* safe* (synonyms for the topic)) OR (health outcome* (synonyms for the topic)) AND (organisation* climate*) OR (organisation* structure* (synonyms for the topic)) OR (hospital lead* (synonyms for the topic)) OR (hospital manage* (synonyms for the topic)) OR (hospital team* (synonyms for the topic)) OR (hospital serv* (synonyms for the topic)) OR (caregiv* (synonyms for the topic)) OR (hospital environment* (synonyms for the topic)) AND (hospital (population)) AND (adult*) AND NOT (children) AND NOT (outpatient*)
