# Supplementary material for: Towards a Quality Care Climate Perspective: A Systematic Review of Associations Among Patient Experience, Patient Outcomes, and Organisational Climate Factors in Hospitals
Source: Int J Environ Res Public Health. 2026 Feb 20;23(2):268. doi: 10.3390/ijerph23020268 (PMC12940218; doi:10.3390/ijerph23020268)
Supplement: Supplementary file 1 [file ijerph-23-00268-s001.zip › Supplementary D. Interactive mapping of study contexts.pdf]

Figure 3 Interactive mapping of study contexts, research designs, and variables

See link below:

https://eppi.ioe.ac.uk/cms/Portals/35/Maps/stavanger_patient_experience.html
